# Granzyme B-activated IL18 potentiates αβ and γδ CAR T cell immunotherapy in a tumor-dependent manner

**DOI:** 10.1016/j.ymthe.2024.05.013

**Published:** 2024-05-14

**Authors:** Caroline M. Hull, Daniel Larcombe-Young, Roberta Mazza, Molly George, David M. Davies, Anna Schurich, John Maher

**Affiliations:** 1 Leucid Bio Ltd, Guy’s Hospital, Great Maze Pond, London SE1 9RT, UK; 2 King’s College London, School of Cancer and Pharmaceutical Sciences, CAR Mechanics Lab, Guy’s Cancer Centre, Great Maze Pond, London SE1 9RT, UK; 3 King’s College London, Department of Infectious Diseases, School of Immunology and Microbial Sciences, Guy’s Hospital, Great Maze Pond, London SE1 9RT, UK; 4 Department of Immunology, Eastbourne Hospital, Kings Drive, Eastbourne, East Sussex BN21 2UD, UK

**Keywords:** Chimeric antigen receptor, αβ T cell, γδ T cell, granzyme B, IL18, cancer, immunotherapy, CAR T-cell

## Abstract

Interleukin (IL)18 is a potent pro-inflammatory cytokine that is activated upon caspase 1 cleavage of the latent precursor, pro-IL18. Therapeutic T cell armoring with IL18 promotes autocrine stimulation and positive modulation of the tumor microenvironment (TME). However, existing strategies are imperfect since they involve constitutive/poorly regulated activity or fail to modify the TME. Here, we have substituted the caspase 1 cleavage site within pro-IL18 with that preferred by granzyme B, yielding GzB-IL18. We demonstrate that GzB-IL18 is constitutively released but remains functionally latent unless chimeric antigen receptor (CAR) T cells are activated, owing to concomitant granzyme B release. Armoring with GzB-IL18 enhances cytolytic activity, proliferation, interferon (IFN)-γ release, and anti-tumor efficacy by a similar magnitude to constitutively active IL18. We also demonstrate that GzB-IL18 provides a highly effective armoring strategy for γδ CAR T cells, leading to enhanced metabolic fitness and significant potentiation of therapeutic activity. Finally, we show that constitutively active IL18 can unmask CAR T cell-mediated cytokine release syndrome in immunocompetent mice. By contrast, GzB-IL18 promotes anti-tumor activity and myeloid cell re-programming without inducing such toxicity. Using this stringent system, we have tightly coupled the biological activity of IL18 to the activation state of the host CAR T cell, favoring safer clinical implementation of this technology.

## Graphical abstract

**Figure:**
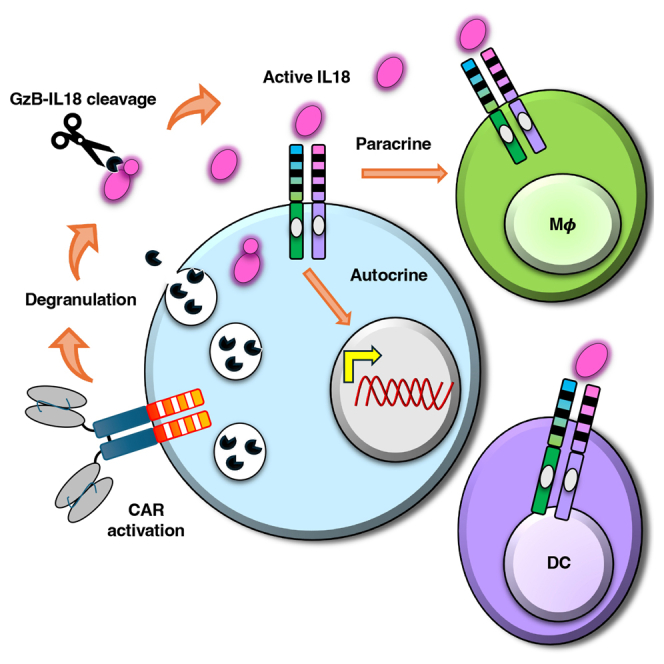

## Teaser

Maher and colleagues describe a granzyme B-activated form of IL18 (GzB-IL18) that provides a safer solution to CAR T cell armoring with this pro-inflammatory cytokine. GzB-IL18 only undergoes cleavage upon encounter with extracellular granzyme B, thereby tightly coupling its biological activity to CAR T cell activation.

## Introduction

Although highly effective against selected hematological malignancies, chimeric antigen receptor (CAR) T cell immunotherapy has poor efficacy against solid tumors. To address this, cytokine armoring technologies have been widely employed.^1^ One attractive option entails engineering of the cells to produce interleukin (IL)18. IL18 is synthesized as an inactive precursor (pro-IL18) that acquires full pro-inflammatory biological function when the N-terminal pro-peptide is proteolytically removed by the cytosolic enzyme, caspase 1.^2^ Mature IL18 signals via MyD88^3^ to facilitate T helper (Th)1-polarized immune responses in which early effector CD8^+^ cytotoxic T cells^4^ are potentiated through enhanced CD4^+^ T cell help.^5^ In the context of cancer, IL18 enables the recruitment of tumor-infiltrating lymphocytes,^6^^,^^7^ natural killer (NK) cells,^8^ dendritic cells (DCs),^8^ and M1-polarized macrophages^4^ while reducing regulatory T cells and M2-polarized macrophages.^4^ IL18 also enhances the expansion and intrinsic anti-tumor activity of NK^9^ and γδ T cells,^10^ innate cell types with attractive properties for allogeneic CAR-based immunotherapy.^11^^,^^12^^,^^13^

IL18 armoring systems generally express the active form of this cytokine in conventional (mainly αβ) T cells, leading to enhanced anti-tumor efficacy^4^^,^^5^ even without lymphodepletion.^14^ This is accompanied by favorable modulation of the tumor microenvironment (TME)^4^^,^^14^ and amplification of endogenous immune surveillance via epitope spreading.^14^

However, the establishment of a constitutive IL18 autocrine loop in CAR T cells presents safety concerns due to the potential for uncontrolled proliferation and/or stimulation of the T cells. Although IL18 has proved relatively non-toxic in human clinical trials,^15^^,^^16^ it has been linked to severe inflammatory disease, particularly when the ratio between active IL18 and IL18-binding protein (its natural regulator) are distorted.^17^^,^^18^^,^^19^^,^^20^ Levels of active IL18 correlate with disease severity in secondary hemophagocytic syndrome,^21^ which is an established complication of CAR T cell immunotherapy.^22^ CAR T cells that constitutively release IL18 have also caused toxicity in immune-competent mouse models.^5^ Consequently, efforts have been made to regulate IL18 release in an activation-dependent manner, thereby conferring tumor-dependent spatiotemporal control over its biological activity.^4^ In one approach, IL18 is expressed under the control of a nuclear factor of activated T cells (NFAT) promoter.^23^^,^^24^ Using this solution, pg/mL levels of IL18 are produced in the activated state, leading to local recruitment of monocytes and NK cells.^25^ However, intrinsic cytolytic activity of the CAR T cells was not enhanced and *in vivo* functionality of this system remains untested.^24^^,^^25^ Moreover, NFAT-regulated expression of the related cytokine, IL12, was insufficiently stringent when evaluated clinically to avoid unacceptable systemic toxicity.^26^ Alternatively, the release of granulocyte macrophage colony stimulating factor (GM-CSF) by activated CAR T cells has been harnessed to achieve autocrine stimulation of a GM-CSF/IL18 receptor heterodimer pair. However, this solution does not elicit the immunomodulatory effects of IL18 on other cell types within the TME.^27^

In this study, we set out to armor CAR T cells with a form of latent IL18 that is selectively activated upon CAR engagement. To achieve this, we substituted the caspase 1 cleavage site found in pro-IL18 with a modification that instead allows proteolytic activation by granzyme B (dubbed GzB-IL18). We hypothesized that GzB-IL18 would only encounter active GzB extracellularly following CAR T cell-mediated degranulation, leading to cleavage and local activation of this cytokine. We report that GzB-IL18 demonstrates biological activity that is strictly dependent on T cell activation, thereby potentiating the anti-tumor activity of CAR-engineered αβ or γδ T cells in an antigen-dependent manner. Furthermore, we demonstrate the potential for toxicity related to constitutive IL18 activity in immunocompetent mice, a finding that is not induced by GzB-IL18.

## Results

### Engineering CAR T cells to co-express granzyme B-cleavable IL18

**Figure 1:**
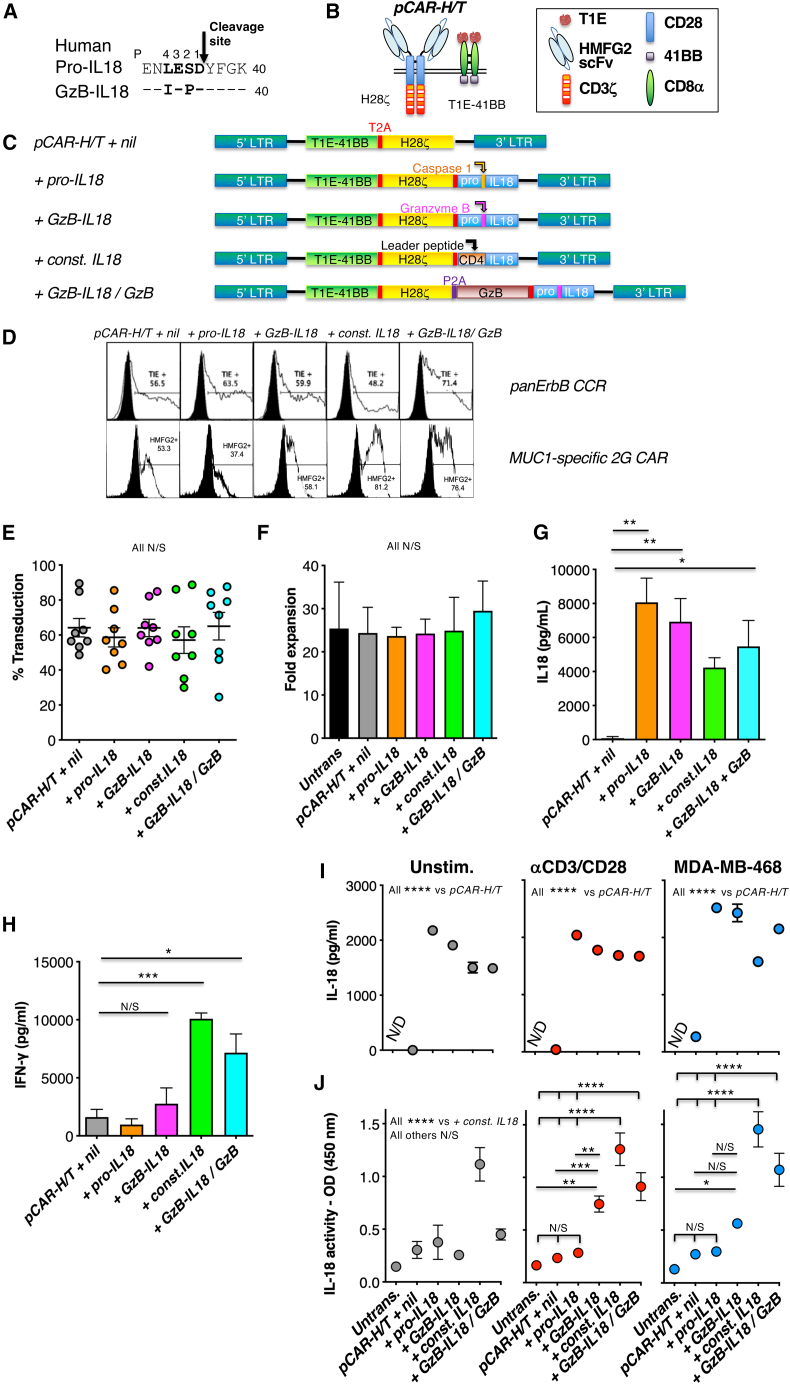
Co-expression of IL18 variants with *pCAR-H/T* (A) The caspase 1 cleavage site in human pro-IL18 (P1–4; shown in bold) is aligned above a mutated variant, GzB-IL18, in which this sequence has been replaced with an optimized human granzyme B (GzB)-cleavage site. (B) Cartoon structure of the MUC1-specific parallel CAR, *pCAR-H/T*. The HMFG2 single-chain variable fragment (scFv) binds to underglycosylated (tumor-associated) MUC1. The T1E peptide binds to eight of nine ErbB homo- and heterodimers. (C) SFG retroviral vectors that encode for *pCAR-H/T* alone (designated as *+ nil*) or *pCAR-H/T + pro-IL18*, *+ GzB-IL18*, *+ constitutively active (const.) IL18* or *+ GzB-IL18/GzB*. Constitutively active IL18 was generated by placing the mature IL18 sequence downstream of a CD4 signal peptide. In the *+ GzB-IL18/GzB* construct, additional GzB is co-expressed with GzB-IL18 and *pCAR-H/T*. GzB and caspase 1 cleavage sites are indicated. LTR, long terminal repeat. (D) Representative examples of pCAR expression by T cells transduced with the vectors shown in (C). (E) Transduction efficiency of replicate donors as determined by flow cytometric analysis of surface CCR expression (mean ± SEM; *n* = 8). All not significant (N/S) by one-way ANOVA. (F) Fold expansion of each CAR T cell population over 10 days (mean ± SEM; *n* = 4). All N/S by one-way ANOVA. (G) IL18 was measured by ELISA in supernatants harvested from the indicated transduced T cell populations after expansion for 10 days (mean ± SEM, *n* = 4 donors). ∗*p* < 0.05; ∗∗*p* < 0.01 by one-way ANOVA. (H) IFN-γ was measured by ELISA in supernatants harvested from the indicated transduced T cell populations following expansion for 10 days (mean ± SEM, *n* = 2 donors measured in duplicate). ∗*p* < 0.05; ∗∗∗*p* < 0.001; N/S, not significant by one-way ANOVA. (I) T cells were transduced with the indicated retroviral vectors, or untransduced (untrans.) as control. T cells were plated at a density of 5 × 10^5^/mL and cultured alone, co-cultured with MDA-MB-468 cells (at a ratio of 10 to 1), or co-cultured with anti-CD3/CD28 TransAct beads. Supernatants were collected after 24 h and analyzed for IL18 by ELISA (mean ± SEM, *n* = 3 donors measured in triplicate). ∗∗∗∗*p* < 0.0001 by two-way ANOVA. (J) Supernatants described in (B) were added to HEK-Blue IL18 reporter cells to assess IL18 biological activity, measured as optical density (OD) at 450 nm (mean ± SEM, n = 3–8 donors measured in triplicate). ∗∗∗∗*p* < 0.0001, ∗∗∗*p* < 0.001, ∗∗*p* < 0.01, ∗*p* < 0.05 by two-way ANOVA.

Cysteine aspartic proteases (caspases) cleave substrate polypeptides following recognition of a four-amino-acid sequence motif (P1–P4) in which aspartic acid (D) is strongly preferred at the P1 position. Granzyme B also possesses a similar preference for D in the P1 position.^28^ Consequently, to engineer GzB-activated human IL18 (GzB-IL18), we mutated P1–P4 within IL18 to a tetrapeptide that is strongly preferred by human GzB (Figure 1A).^29^^,^^30^ To test GzB-IL18 function, it was co-expressed in conventional (mainly αβ) T cells with a parallel (p)CAR named *pCAR-H/T*.^31^ *pCAR-H/T* consists of a MUC1-specific CD28-CD3ζ CAR^32^ co-expressed with a chimeric co-stimulatory receptor (CCR) in which the pan-ErbB ligand, T1E (a chimeric peptide derived from transforming growth factor α and epidermal growth factor),^33^ is coupled to 4-1BB (Figure 1B). This arrangement maintains MUC1 specificity of tumor targeting. However, when ErbB dimers are also present, cytolytic activity, cytokine release, and anti-tumor activity are further enhanced via optimized dual CD28/4-1BB co-stimulation.^31^ Structure of the *pCAR-H/T + GzB-IL18* retroviral vector is shown in Figure 1C, together with controls in which pro-IL18 (*+pro-IL18*) or constitutively active IL18 (*+const. IL18*) are co-expressed. Constitutively active IL18 was engineered by fusion of active IL18 downstream of a CD4 leader peptide. To determine whether GzB levels are a limiting factor for GzB-IL18 function, we also co-expressed additional GzB with GzB-IL18 and *pCAR-H/T* (dubbed *+GzB-IL18/GzB*). Representative examples of flow cytometric analysis of these cells are shown (Figure 1D) together with replicates that demonstrate comparable transduction efficiency across groups (Figure 1E). Expression of these IL18 variants had no effect on CAR T cell expansion (Figure 1F). Large amounts of IL18 were detected by ELISA in derived supernatants, irrespective of the cleavage site present (Figure 1G). The data are consistent with correct expression and folding of these IL18-based proteins.

### Analysis of functional activity of GzB-IL18

IL18 was originally identified as an interferon (IFN)-γ inducing factor.^34^ Consequently, we analyzed levels of IFN-γ present in these cultures after 14 days. As expected, *+const. IL18* cultures contained very high levels of IFN-γ (Figure 1H). By contrast, levels of IFN-γ found in *+pro-IL18* and *+GzB-IL18* cultures were similar to *pCAR-H/T + nil* cultures (Figure 1H). In *+GzB-IL18/GzB* cultures, IFN-γ levels also increased significantly (Figure 1H). These data indicate that the IFN-γ-inducing ability of GzB-IL18 is negligible in non-activated CAR T cells unless GzB is co-expressed.

Next, we compared the release of immunochemical (e.g., ELISA detectable) and biologically active IL18 by these cells. CAR T cells were activated using CD3+CD28-coated beads or co-culture with (MUC1^+^ ErbB^+^) MDA-MB-468 triple-negative breast cancer (TNBC) cells, making comparison with unstimulated controls. High concentrations of IL18 were detected by ELISA in *+pro-IL18*, *+GzB-IL18*, *+const. IL18*, or *+GzB-IL18/GzB* cultures irrespective of activation status, in contrast to untransduced or *pCAR-H/T + nil* controls (Figure 1I). To compare IL18 biological activity, supernatants were added to HEK-Blue IL18 reporter cells (Figure 1J). As expected, pro-IL18 was biologically inactive, while const. IL18 demonstrated high activity, irrespective of activation status. In the case of GzB-IL18, negligible activity was evident when cells were unstimulated. Biological activity increased significantly when *+GzB-IL18* T cells were stimulated using CD3+CD28 crosslinking, or more modestly when CAR T cells were co-cultured on MDA-MB-468 TNBC monolayers. When GzB was co-expressed with GzB-IL18 (*+GzB-IL18/GzB*), IL18 bioactivity was significantly increased in both activation conditions.

To confirm CAR-dependent processing of GzB-IL18, GzB-IL18 was co-expressed with a control pCAR in which CD3ζ sequences were removed from the CAR endodomain (*trunc.pCAR-H/T*; Figure S1A). No IL18 biological activity was detected when *trunc.pCAR-H/T + GzB-IL18* T cells were co-cultured with MDA-MB-468 tumor cells (Figure S1B). By contrast, CD3+CD28 crosslinking did induce IL18 biological activity in supernatant collected from these T cells (Figure S1B). Also as expected, biologically active IL18 was detected when T cells expressing *pCAR-H/T + GzB-IL18* were stimulated either on MDA-MB-468 tumor monolayers or by CD3+CD28 crosslinking (Figure S1B). Moreover, co-culture of *pCAR-H/T + GzB-IL18* T cells with MUC1-negative MDA-MB-435 cells did not result in the release of biologically active IL18, in contrast to *pCAR-H/T + const. IL18* T cells (Figure S1C). Taken together, these data demonstrate that induction of GzB-IL18 biological activity is stringently linked to activation of the host T cell.

### *In vitro* anti-tumor activity of *pCAR-H/T + nil* CAR T cells is potentiated by GzB-IL18

**Figure 2:**
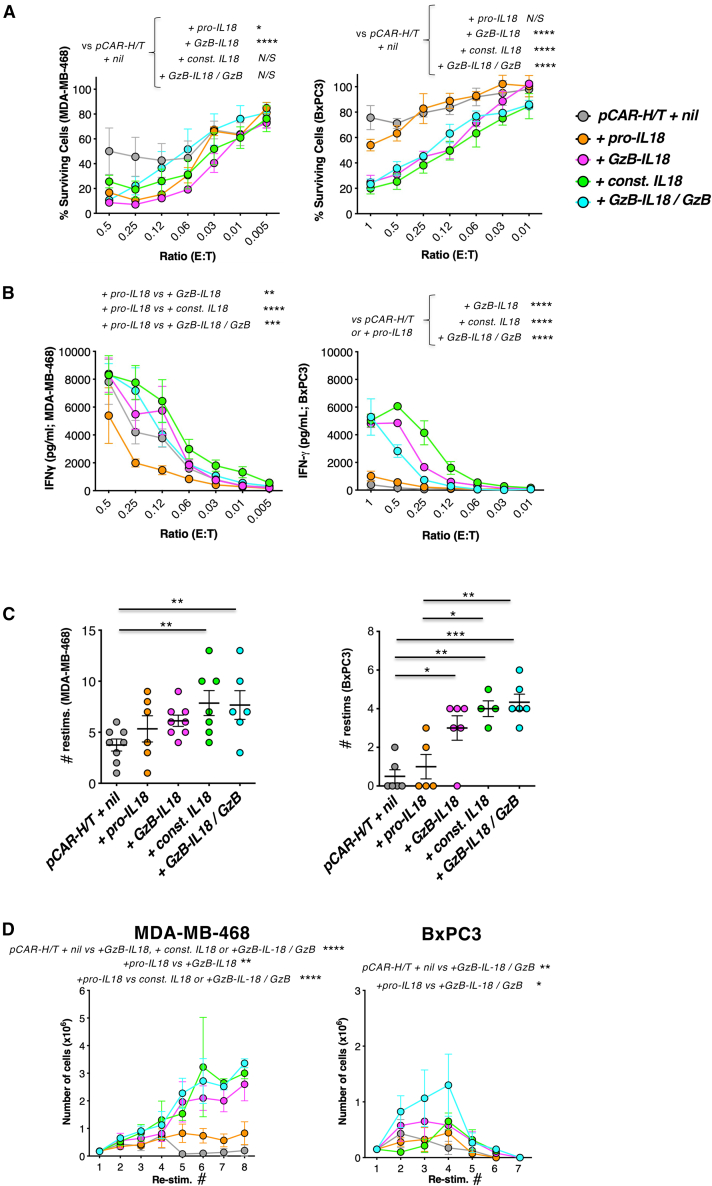
GzB-IL18 promotes CAR T cell anti-tumor activity *in vitro* (A) Cytotoxicity assays were conducted with MDA-MB-468 TNBC (left) or BxPC3 pancreatic cancer cells (right), which were incubated for 72 h with the indicated CAR T cell populations at the specified effector:target (E:T) ratio (mean ± SEM, *n* = 5). After removal of residual T cells, tumor cell viability was measured using an MTT assay. ∗∗∗∗*p* < 0.0001, ∗*p* < 0.05 by two-way ANOVA. (B) IFN-γ concentration was measured in supernatants harvested after 72 h from co-cultures described in (A) (mean ± SEM, *n* = 5). ∗∗*p* < 0.01, ∗∗∗*p* < 0.001, ∗∗∗∗*p* < 0.0001 by two-way ANOVA. (C) CAR T cells were added to MDA-MB-468 (left) or BxPC3 tumor cells (right) at a 1:1 E:T ratio (1 × 10^4^ tumor cells). Tumor viability was determined after 72 h and T cells were transferred to a fresh well containing 1 × 10^4^ tumor cells. T cells were re-stimulated in this manner until they could no longer be retrieved from tumor monolayers. A stimulation cycle was deemed successful if ≥60% of tumor cells were destroyed. The number of successful re-stimulation cycles for each T cell/tumor cell condition is shown (mean ± SEM; n = 4–8 donors measured in triplicate). ∗∗∗*p* < 0.001, ∗∗*p* < 0.01, ∗*p* < 0.05 by two-way ANOVA. (D) T cell number was determined prior to each re-stimulation cycle, undertaken as described in (C) (mean ± SEM, n = 3–7 donors). ∗∗∗∗*p* < 0.0001, ∗∗*p* < 0.01, ∗*p* < 0.05 by two-way ANOVA.

We first assessed the functional impact of IL18 armoring using conventional (predominantly αβ) CAR T cells. Anti-tumor activity at low effector to target (E:T) ratios was compared in co-cultures with MDA-MB-468 cells and BxPC3 pancreatic tumor cells (also MUC1^+^ ErbB^+^), which respectively are sensitive and moderately resistant to CAR T cell killing.^35^ Cytotoxic activity of *pCAR-H/T* was potentiated by GzB-IL18 to a comparable degree to that seen with const. IL18 (Figure 2A). Provision of additional GzB (*+GzB-IL18/GzB*) did not further enhance activity. Significantly greater production of IFN-γ was also observed in *+GzB-IL18*, *+const. IL18*, and *+GzB-IL18/GzB* tumor co-cultures (Figure 2B). However, this was not observed in *+pro-IL18* cultures, indicating that pro-IL18 remains inactive despite CAR stimulation.

Next, we undertook tumor re-stimulation experiments to assess whether these formats of IL18 delay the onset of functional exhaustion, indicated by failure to destroy ≥60% of tumor cells within each 72-h re-stimulation cycle. Productive re-stimulation cycle number was increased in *+const. IL18* and *+GzB-IL18/GzB* cultures, while a similar trend was observed with *+GzB-IL18* CAR T cells (Figure 2C). This was accompanied by enhanced T cell expansion (Figure 2D) and sustained IFN-γ production during each stimulation cycle (Figure S2). To confirm that functional activity of IL18 was dependent on CAR activation, we compared the re-stimulation potential of *trunc.pCAR-H/T + GzB-IL18* and *pCAR-H/T + GzB-IL18* T cells. As expected, tumor monolayers were only destroyed and T cell proliferation only evident in *pCAR-H/T + GzB-IL18* T cell co-cultures (Figure S1D), confirming that CAR-dependent activation was required for these activities.

To further understand the pro-inflammatory impact of GzB-IL18, we co-cultured *pCAR-H/T* T cells and their armored derivatives with MDA-MB-468 tumor cells (Figure S3A) or anti-CD3+CD28 beads (Figure S3B) and measured tumor necrosis factor (TNF)-⍺ and IL2 in derived supernatants. T cells armored with GzB-IL18 produced significantly more TNF-⍺ than those with pro-IL18 or no armoring when stimulated on tumor cell monolayers or with CD3+CD28 crosslinking (Figure S3). GzB-IL18-armored T cells produced significantly more IL2 in response to CD3+CD28 crosslinking, but not tumor cells (Figure S3).

To confirm these findings using a different CAR, GzB-IL18 was co-expressed with a pan-ErbB-specific CAR, *2G-T*,^31^^,^^36^ in which targeting is achieved using the T1E peptide^33^ (Figures S4A and S4B). Inclusion of GzB-IL18 did not alter transduction efficiency (Figures S4C and S4D) and IL18 could once again be detected by ELISA analysis of *2G-T + GzB-IL18* T cell supernatants, irrespective of CAR T cell activation status (Figure S4E). However, biologically active IL18 was only detected when *2G-T + GzB-IL18* CAR T cells were activated via the CAR or anti-CD3+CD28 crosslinking (Figure S4F). GzB-IL18 enhanced re-stimulation capacity on MDA-MB-468 or BxPC3 tumor monolayers (Figure S4G), accompanied by increased CAR T cell expansion (Figure S4H) and sustained IFN-γ production (Figure S4I). These data confirm that GzB-IL18 delivers stringent, activation-dependent IL18 function.

### *In vivo* anti-tumor activity of *pCAR-H/T* CAR T cells is potentiated by GzB-IL18

**Figure 3:**
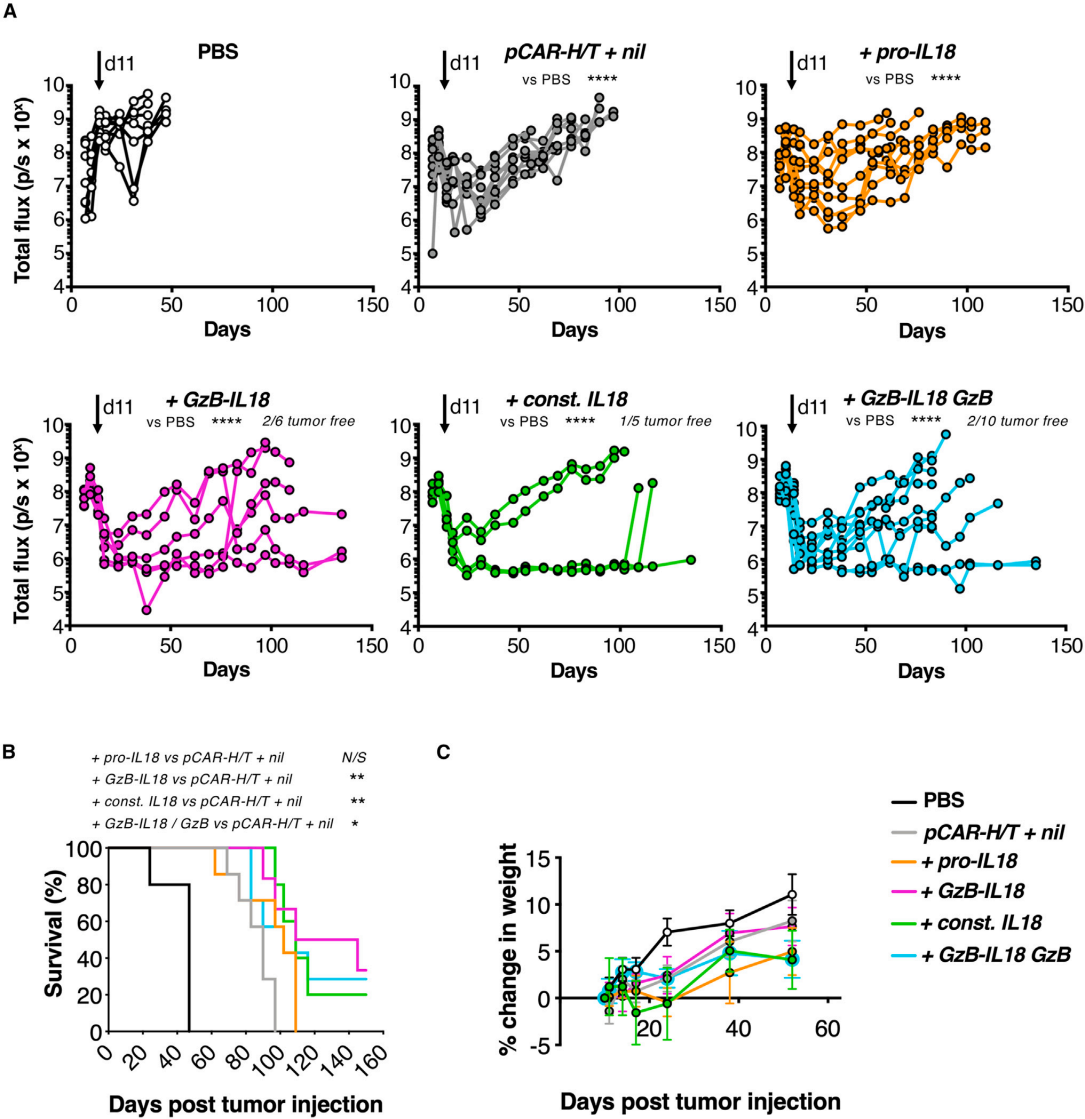
GzB-IL18 promotes CAR T cell anti-tumor activity *in vivo* (A) 1 × 10^6^ ffLuc-expressing MDA-MB-468 tumor cells were injected i.p. into female SCID Beige mice. After confirmation of tumor engraftment using BLI, mice were randomly assorted into groups with similar mean tumor burden. Animals received a single dose of 10 × 10^6^ of the indicated CAR T cells i.p. or PBS as control on day 11 (overhead arrow). Plots indicate serial bioluminescence emission from each mouse. ∗∗∗∗*p* < 0.0001 by two-way ANOVA. Number of tumor-free mice at the end of the experiment is indicated. (B) Survival curve of mice treated as described in (A). Statistical analysis was by log rank (Mantel-Cox) test: ∗∗*p* < 0.01; ∗*p* < 0.05; N/S, not significant. (C) Weight of mice treated as described in (A) (mean ± SEM, n = 5–7).

To test *in vivo* anti-tumor activity, an intraperitoneal (i.p.) TNBC xenograft model was established in severe combined immunodeficiency (SCID) Beige mice using firefly luciferase (ffLuc)-expressing MDA-MB-468 cells. Once tumor was established for 11 days, animals were treated with i.p. *pCAR-H/T + nil* CAR T cells or IL18-armored derivatives. Disease burden was monitored by serial bioluminescence imaging (BLI; Figure 3A). Treatment with *pCAR-H/T + nil* CAR T cells resulted in short-lived tumor control followed by relapse and this was not improved by armoring with pro-IL18. In contrast, sustained disease control was observed in some mice following treatment with *+GzB-IL18*, *+const. IL18*, and *+GzB-IL18/GzB* T cells, with no difference in efficacy observed between these three groups. Survival of mice in all three groups was significantly prolonged compared to the *pCAR-H/T + nil* control group (Figure 3B). Following treatment with all CAR T cells, mice continued to gain weight (Figure 3C), indicating that toxicity was not evident, although there is limited understanding of the biological activity of human IL18 in the mouse. Together, these data demonstrate that GzB-IL18 armoring enhances CAR T cell anti-tumor efficacy *in vivo*.

### *In vitro* anti-tumor activity of *pCAR-H/T* engineered γδ T cells is potentiated by GzB-IL18

**Figure 4:**
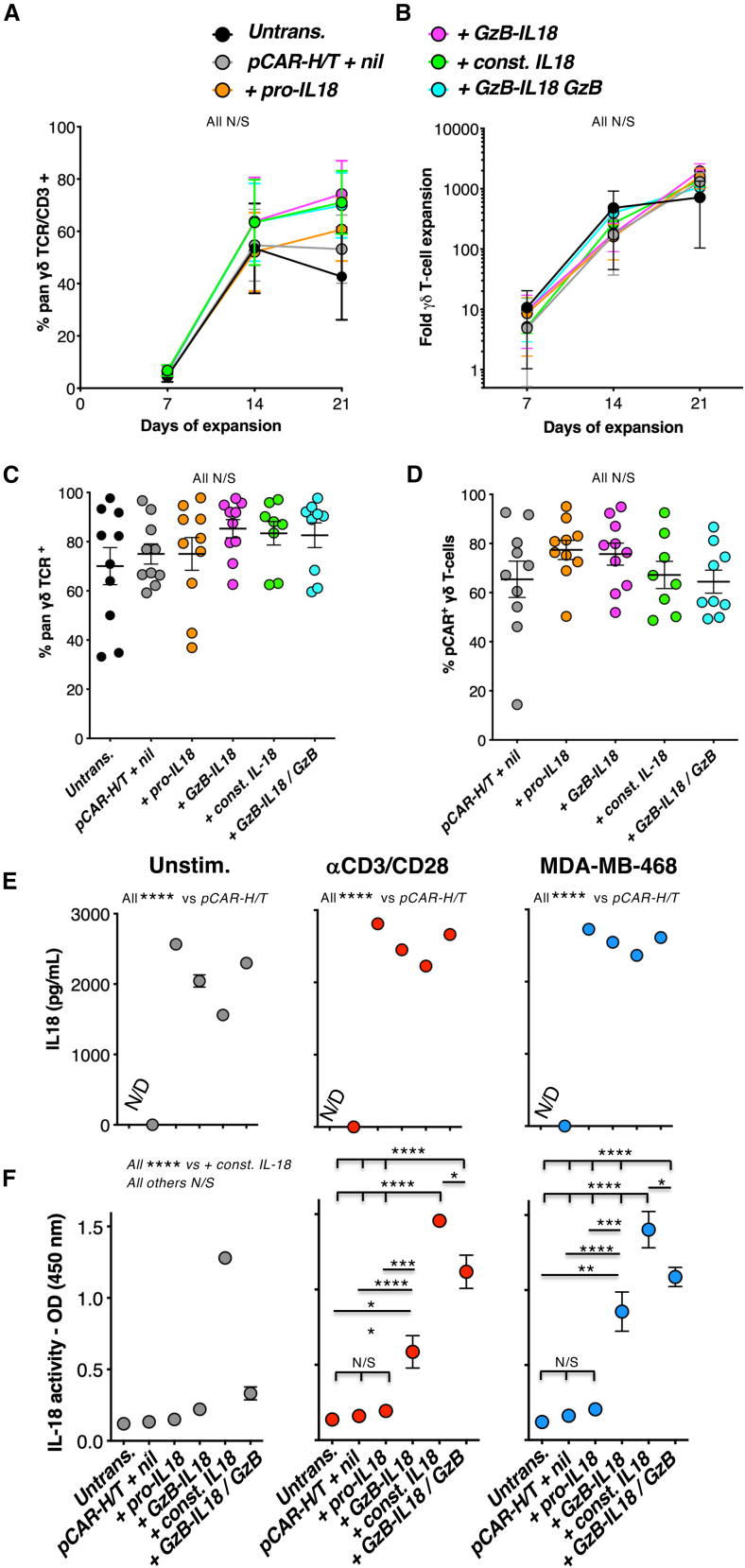
GzB-IL18 promotes γδ CAR T cell function *in vitro* (A) Flow cytometric analysis of γδ T cells engineered to express the indicated transgenes. γδ TCR expression was determined using a pan-γδ TCR antibody (mean ± SEM, n = 4–5 donors). All N/S by two-way ANOVA. (B) Fold expansion of the indicated γδ T cells over 21 days of culture is shown (mean ± SEM, *n* = 4 donors). Untrans., untransduced. All N/S by two-way ANOVA. Percentage γδ T cell purity (C) and percentage γδ T cells that co-express *pCAR-H/T* (D) are shown for replicate cultures on day 21 of expansion (mean ± SEM, n = 8–10 donors). All N/S by one-way ANOVA. (E) γδ T cells were transduced with the indicated retroviral vectors or untransduced as control. γδ T cells were plated at a density of 5 × 10^5^/mL and cultured alone, co-cultured with MDA-MB-468 cells (at a ratio of 10 to 1), or co-cultured with anti-CD3/CD28 TransAct beads. Supernatants were collected after 24 h and analyzed for IL18 by ELISA (mean ± SEM, *n* = 3 donors measured in triplicate). ∗∗∗∗*p* < 0.0001 by two-way ANOVA. (F) Supernatants described in (E) were added to HEK-Blue IL18 reporter cells to assess IL18 biological activity, measured as OD at 450 nm (mean ± SEM, n = 2–6 donors measured in triplicate). ∗∗∗∗*p* < 0.0001, ∗∗∗*p* < 0.001, ∗∗*p* < 0.01, ∗*p* < 0.05 by two-way ANOVA.

γδ T cells offer great promise as a potential off-the-shelf cancer cell therapy chassis. When circulating γδ T cells are expanded in the presence of transforming growth factor (TGF)-β, their intrinsic anti-tumor activity is potentiated.^37^ Moreover, TGF-β-educated γδ T cells demonstrate enhanced anti-tumor activity when engineered to express *pCAR-H/T*.^13^ Given that IL18 has also been reported to boost anti-tumor activity of human γδ T cells,^10^ we next set out to test whether GzB-IL18 armoring could be used to potentiate *pCAR-H/T*^+^ γδ T cells. Following γδ T cell activation and retroviral transduction, percentage of CAR-expressing γδ T cells progressively increased (Figures 4A and S5A), accompanied by exponential expansion in response to IL2 and TGF-β (Figure 4B). Both Vδ2 and Vδ1 cells were present with, as expected, a greater proportion of the former (Figure S5B). Cells had a mainly central and effector memory phenotype, with a trend toward greater differentiation when constitutive IL18 was produced (Figure S5C). γδ T cell purity (Figure 4C) and transduction efficiency (Figure 4D) were comparable across groups.

Activation-dependent induction of IL18 function was next evaluated in CAR γδ T cells. Figure 4E shows that all armored CAR γδ T cell populations secrete IL18 that is detectable by ELISA, independent of activation state. Following anti-CD3+CD28 crosslinking or co-culture with MDA-MB-468 tumor cells, IL18 biological activity was significantly elevated in *+GzB-IL18*-derived supernatants, albeit not to the level seen with *+const. IL18* or *+GzB-IL18/GzB* (Figure 4F).

We next compared these IL18-armored γδ CAR T cells in tumor re-stimulation assays. Co-expression of GzB-IL18 with *pCAR-H/T* promoted a significant (MDA-MB-468) or a trend (BxPC3) toward an increased number of stimulation cycles in which ≥60% of tumor was destroyed (Figure S6A). This was accompanied by increased IFN-γ production, which was significant in the case of MDA-MB-468 cultures (Figure S6B).

To confirm these findings, γδ T cells were engineered to express the *2G-T* pan-ErbB-specific CAR, either alone or with GzB-IL18 (Figures S7A and S7B). Functional activity of IL18 was again dependent upon activation of *2G-T + GzB-IL18* cells (Figure S7C). GzB-IL18-armored *2G-T* CAR T cells achieved greater tumor re-stimulation capacity (Figure S7D) accompanied by enhanced IFN-γ production (Figure S7E) and a trend toward increased CAR T cell expansion (Figure S7F) upon repeated tumor cell exposure.

### *In vivo* anti-tumor activity of *pCAR-H/T* γδ T cells is potentiated by GzB-IL18

**Figure 5:**
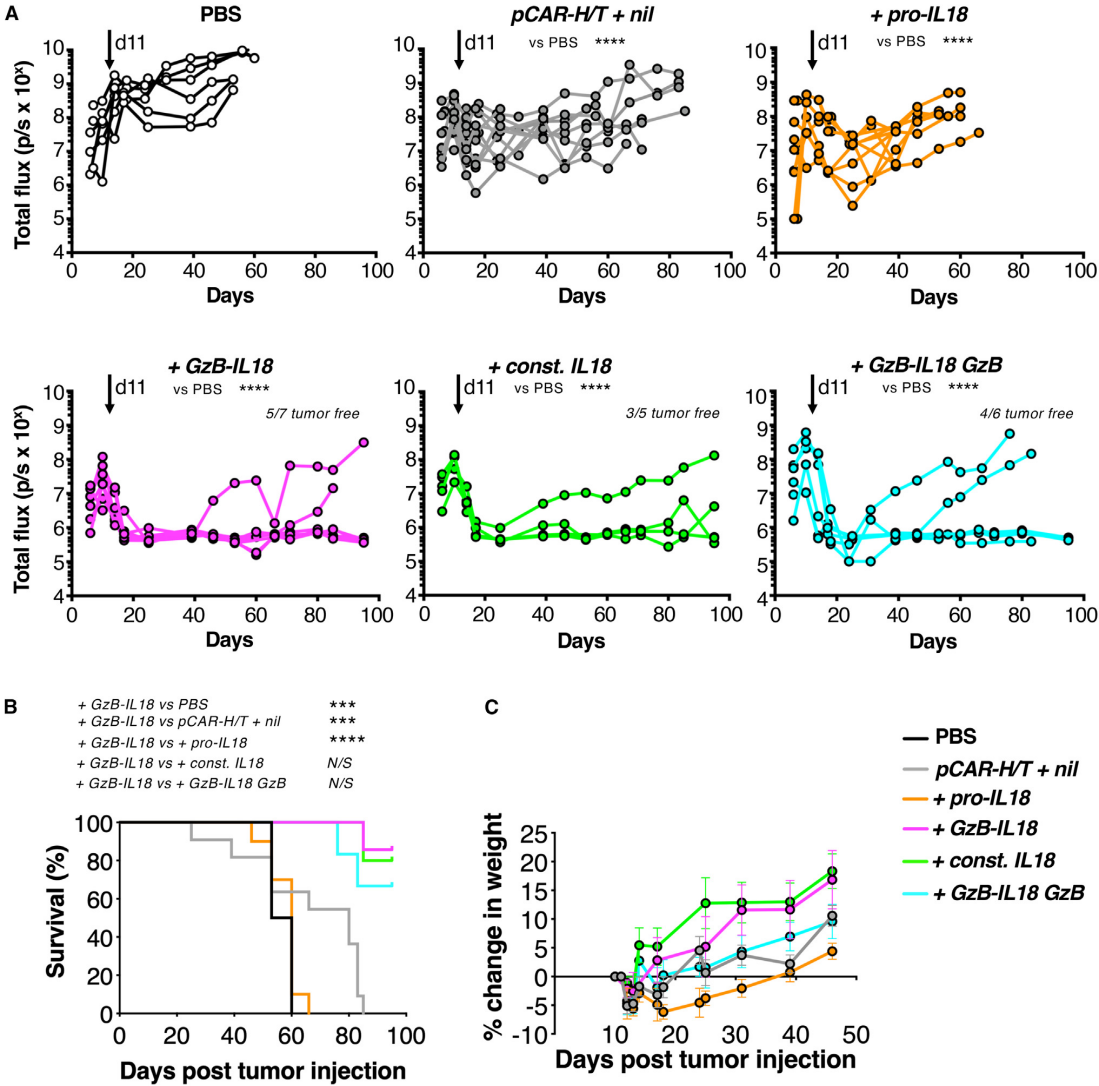
GzB-IL18 promotes γδ CAR T cell function *in vivo* (A) 1 × 10^6^ ffLuc-expressing MDA-MB-468 tumor cells were injected i.p. into SCID Beige mice. After confirmation of tumor engraftment using BLI, mice were randomly assorted into groups with similar mean tumor burden. Animals received a single dose of 10 × 10^6^ of the indicated CAR γδ T cells i.p. or PBS as control on day 11 (overhead arrow). Plots indicate serial bioluminescence emission from each mouse. Number of tumor-free mice at the end of the experiment is indicated. ∗∗∗∗*p* < 0.0001 by two-way ANOVA. (B) Survival curve of mice treated as described in (A). ∗∗∗∗*p* < 0.0001, ∗∗∗*p* < 0.001, ∗∗*p* < 0.01 by log rank (Mantel-Cox) test. (C) Weight of mice treated as described in (A) (mean ± SEM, n = 5–11).

Next, we compared the therapeutic efficacy of IL18-armored γδ CAR T cells in the MDA-MB-468 TNBC xenograft model. Treatment with *pCAR-H/T + nil*-engineered γδ T cells exerted a modest therapeutic effect (Figure 5A), leading to a survival advantage when compared to PBS alone (Figure 5B). No further enhancement of efficacy was observed in the *+pro-IL18* group. By marked contrast, tumor eradication was observed in most mice following treatment with *+GzB-IL18*, *+const. IL18*, or *+GzB-IL18/GzB* CAR T cells, with no difference between these groups observed (Figure 5A). As a result, all three of these groups achieved a significant survival advantage when compared to *pCAR-H/T + nil* or *+pro-IL18* groups (Figure 5B). Following treatment with all CAR T cell products, mice continued to gain weight (Figure 5C). These data demonstrate that +GzB-IL18 armoring also enhances γδ CAR T cell anti-tumor efficacy *in vivo*.

### Metabolic effects of GzB-IL18 on CAR T cells

**Figure 6:**
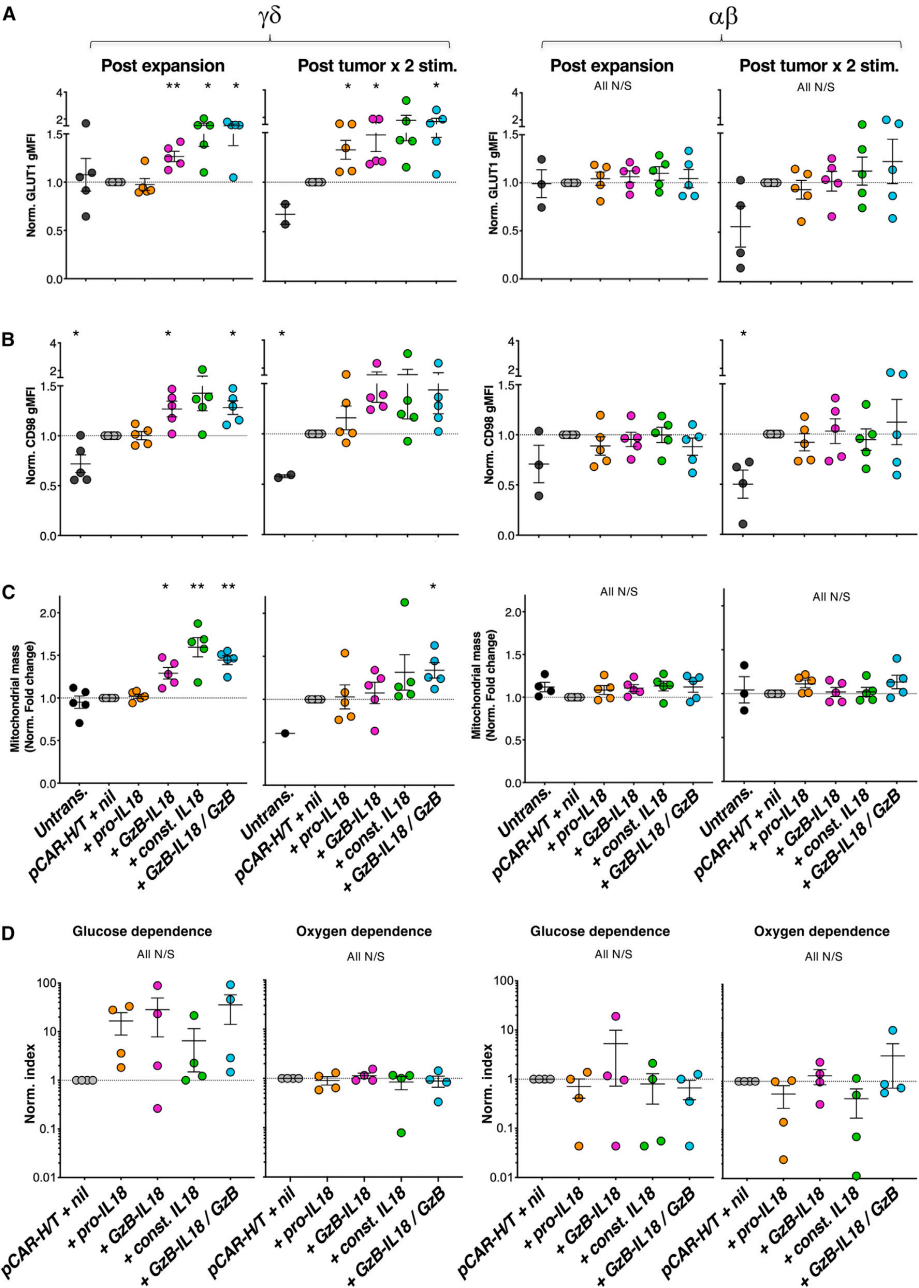
Effects of IL18 armoring on CAR T cell metabolism γδ T cells (left) or conventional (mainly αβ) T cells (right) from *n* = 5 donors were transduced with the indicated constructs and expanded in culture for 3 weeks. GLUT1 (A), CD98 (B), and mitochondrial mass (C) were quantified in CAR T cells by flow cytometry post expansion and post two stimulation cycles with MDA-MB-468 tumor cells. Mean fluorescence intensity of each marker was normalized (norm.) to that of pCAR-H/T *+ nil* T cells, which was set to 1.0 in each experiment. ∗*p* < 0.05, ∗∗*p* < 0.01 using one-sample t test, making comparison with *pCAR-H/T* + nil. (D) Post tumor cell co-culture, CAR T cells were incubated with puromycin for 45 min to determine protein synthesis rate according to the SCENITH method. CAR T cell metabolic pathways were inhibited as described in section “materials and methods” concurrently with puromycin incubation to determine glucose or oxygen dependence. All N/S using one-sample t test.

Given its potentiating effects on anti-tumor activity, particularly in the case of CAR γδ T cells, we next evaluated metabolic effects of IL18 armoring. We first evaluated nutrient transporter expression in IL18-armored αβ and γδ *pCAR-H/T*^*+*^ T cells, either after expansion for 3 weeks or following two additional re-stimulation cycles on MDA-MB-468 tumor cell monolayers. In γδ, but not αβ, T cells, IL18 armoring resulted in upregulation of the glucose transporter, GLUT1 (Figure 6A), and the amino acid transporter, CD98 (Figure 6B). IL18-armored γδ T cells also exhibited an increase in mitochondrial mass, a finding that was not evident in αβ T cells (Figure 6C). Representative examples of these analyses are shown in Figure S8A.

We next evaluated the metabolic phenotype of IL18-armored CAR T cells using single-cell energetic metabolism by profiling translation inhibition (SCENITH). Assays were performed following two cycles of stimulation on MDA-MB-468 tumor monolayers. Although not significant, this analysis demonstrated a trend toward increased glucose-dependent metabolism by IL18-armored γδ CAR T cells when compared to *pCAR-H/T + nil* cells, in line with this metabolic pathway supporting enhanced effector function (Figures 6D and S8B). Once again, this trend was not seen for αβ T cells. No difference was observed for oxygen-dependent metabolism (Figures 6D and S8C) in either T cell subset. We have previously shown that oxygen-dependent metabolism is enhanced by dual CD28 + 4-1BB co-stimulation via the pCAR platform,^31^ and these data confirm that additional IL18 armoring does not further alter this.

### GzB-IL18 but not const. IL18 enhances CAR T cell anti-tumor activity without toxicity in immunocompetent mice

**Figure 7:**
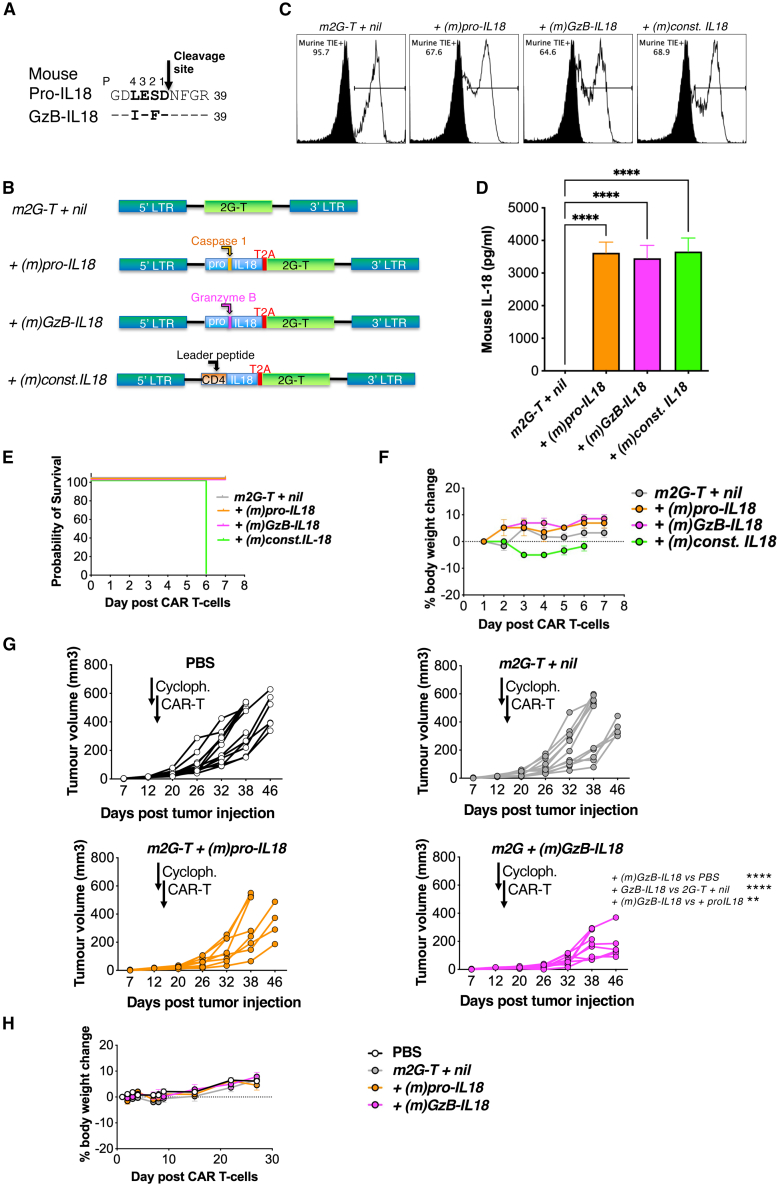
GzB-IL18 safely potentiates anti-tumor activity of panErbB-specific CAR T cell, whereas const. IL18 induces lethal toxicity in this model (A) The caspase 1 cleavage site in mouse pro-IL18 (P1–4; shown in bold) is aligned above a mutated variant, GzB-IL18, in which this sequence has been replaced with an optimized mouse GzB-cleavage site. (B) Murine T cells were transduced using an SFG retroviral vector to express the *m2G-T* panErbB CAR alone or together with the indicated murine IL18 variants. (C) T cell transduction was assessed by staining for murine TIE peptide within the CAR ectodomain at day 5 post transduction. (D) Mouse IL18 was measured by ELISA in supernatant collected from the indicated mouse T cell cultures and plated at 1 million cells/mL for 24 h (mean ± SEM, *n* = 3). ∗∗∗∗*p* < 0.0001 by one-way ANOVA. (E) Survival curve of tumor-free BALB/c mice treated with 4 × 10^6^ mouse CAR T cells (*n* = 3 per group). (F) Weight of mice treated as described in (E) (mean ± SEM, *n* = 3). (G) 1 × 10^6^ B7E3 tumor cells were injected s.c. into female BALB/c mice. After confirmation of tumor engraftment using caliper measurements, mice were randomly assorted into groups with similar mean tumor burden and preconditioned on day 13 with cyclophosphamide (cycloph., first arrow). On day 14 (second arrow), mice received a single dose of 2 × 10^6^ of the indicated mouse CAR T cells i.v., or PBS as control. Plots indicate serial caliper measurements of tumor for each mouse. ∗∗∗∗*p* < 0.0001, ∗∗*p* < 0.01 by two-way ANOVA comparing post-treatment tumor burden. (H) Percentage weight change in mice described in (G) (mean ± SEM, *n* = 8–13).

We next evaluated the safety and impact on efficacy of the mouse versions of these IL18 variants in an immunocompetent mouse tumor model. Balb/c mouse T cells were engineered to co-express a murine panErbB-specific second-generation CAR (*m2G-T*; structure similar to the human version shown in Figure S4A, except that all components were of mouse origin). *m2G-T* was co-expressed with murine (m) variants of pro-IL18, GzB-IL18 and const. IL18. In the case of (m)GzB-IL18, a granzyme B cleavage site that is optimal for the mouse enzyme was selected (Figure 7A).^38^ Structure of retroviral vectors is shown in Figure 7B. Efficient T cell transduction was demonstrated on day 5 post gene transfer (Figure 7C). As expected, mouse IL18 was detected by ELISA in supernatants collected from armored T cell cultures (Figure 7D).

Since panErbB-specific CAR T cells can induce cytokine release syndrome (CRS) in mice,^39^ we initially assessed the safety of these armored CAR T cells in tumor-free animals. In contrast to all other groups, intravenous (i.v.) administration of 4 × 10^6^ *m2G-T + (m)const. IL18* CAR T cells induced sudden and unanticipated lethal toxicity in all mice late on day 6 post T cell infusion, precluding postmortem analysis of carcasses (Figure 7E). This event occurred after a period of mild weight loss in this group (Figure 7F).

Next, an efficacy study was performed in Balb/c mice engrafted with subcutaneous (s.c.) B7E3 head and neck squamous cell carcinoma (HNSCC) tumors. Mice were conditioned on day 13 with cyclophosphamide prior to i.v. treatment on day 14 with 2 × 10^6^ *m2G-T + nil*, *m2G-T + (m)pro-IL18*, or *m2G-T + (m)GzB-IL18* CAR T cells. At this low dose, *m2G-T + nil* CAR T cells had no impact on tumor progression (Figure 7G). Armoring with (m)pro-IL18 marginally improved tumor control compared to *m2G-T + nil*; however, differences were not significant (Figure 7G). In contrast, treatment with *m2G-T + (m)GzB-IL18* T cells significantly reduced tumor progression compared to *m2G-T + nil* cells (Figure 7G) and did not induce clinically evident toxicity or weight loss (Figure 7H). These data indicate that armoring with a murine version of GzB-IL18 enhances therapeutic activity in a solid-tumor immune-competent mouse model without inducing toxicity.

### Const. IL18 exacerbates CAR T cell-induced CRS

**Figure 8:**
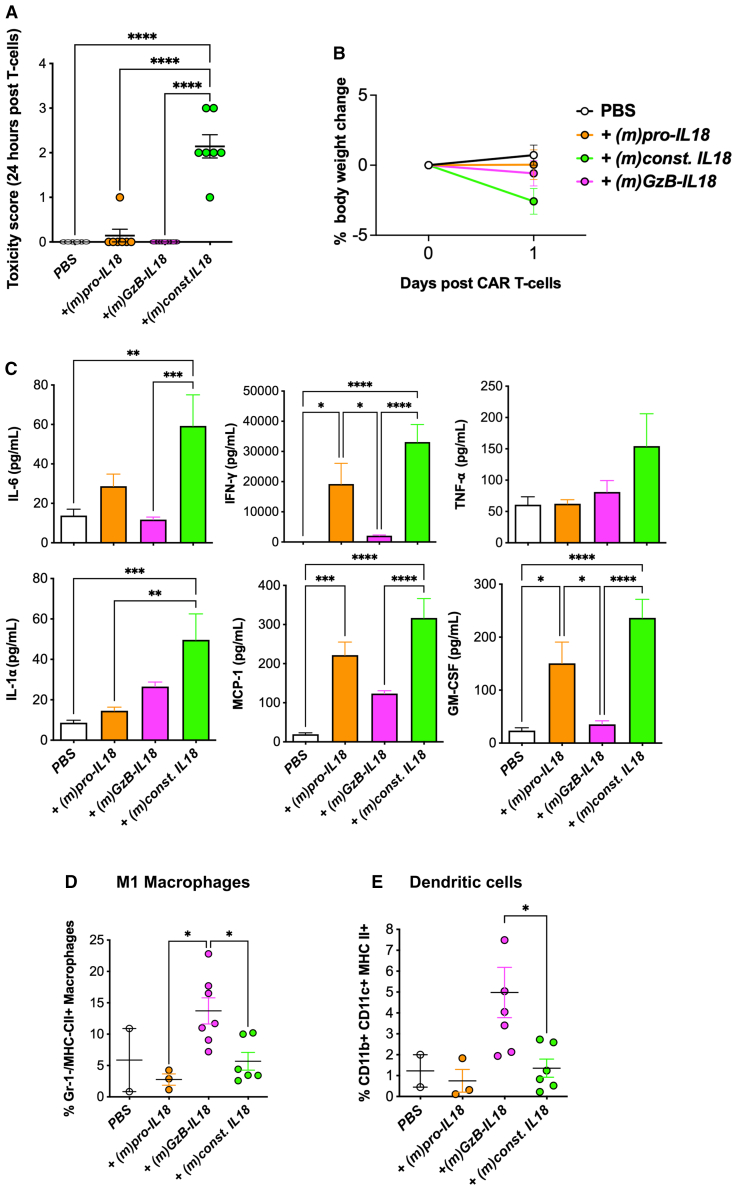
GzB-IL18 polarizes macrophages toward an M1 phenotype, while const. IL18 induces CRS B7E3 tumor cells were engrafted s.c. in Balb/c mice. On day 13, mice were conditioned with cyclophosphamide 50 mg/kg i.p. followed by i.v. infusion of 4 × 10^6^ of the indicated CAR T cell populations on day 14. Data show toxicity scores at 24 h post CAR T cell treatment (mean ± SEM; A) and body weight pre- and 24 h post CAR T cells (mean ± SEM; B). (C) Since toxicity in *m2G-T + (m)const. IL18*-treated mice exceeded humane endpoints, all mice were killed and terminal bleeds performed at 24 h post T cell infusion. Indicated cytokines were measured in derived sera (mean ± SEM, *n* = 11–18 mice). Spleens were also harvested from the mice and subjected to immunophenotyping. (D) M1 macrophages were identified as Lin^−^, CD11b^+^, F4/80^+^, Gr-1^−^, and MHC-II^+^, while (E) dendritic cells (DCs) were identified as CD11b^+^, CD11c^+^, and MHC-II ^+^ (mean ± SEM, n = 2–7). ∗∗∗∗*p* < 0.0001, ∗∗∗*p* < 0.001, ∗∗*p* < 0.01, ∗*p* < 0.05 by one-way ANOVA.

To better understand the toxicity induced by (m)const. IL18 armoring, we performed another experiment in B7E3 tumor-bearing mice, comparing all three IL18-armored variants of *m2G-T* CAR T cells. Mice received 4 × 10^6^ i.v. CAR T cells since this dose had earlier induced lethal toxicity in all mice that had received *m2G-T +(m)const. IL18* T cells. Within 24 h, a significant rise in toxicity score^40^ (Figure 8A) accompanied by weight loss (Figure 8B) was observed in *m2G-T + (m)const. IL18* CAR T cell-treated mice, indicating that humane endpoints had been met. To investigate underlying mechanisms, all animals were immediately exsanguinated under terminal anesthesia and blood was analyzed for a panel of cytokines involved in CRS. Significantly higher concentrations of IL6, IFN-γ, monocyte chemoattractant protein (MCP)-1, and granulocyte macrophage colony stimulating factor (GM-CSF) were detected in blood samples from *m2G-T + (m)const. IL18*-treated mice compared to one or more controls (Figure 8C). By contrast, these cytokines were not elevated in mice treated with *m2G-T + (m)GzB-IL18* CAR T cells (Figure 8C). Although not significant, there was also a trend toward elevated TNFα, IL1α (Figure 8C), IL12, IL17A, IFN-β, and IL23 (Figure S9A) in *m2G-T + (m)const. IL18*-treated mice. Taken together, these data indicate that armoring with (m)const. IL18 had accentuated CRS induced by *m2G-T* CAR T cells, resulting in lethal toxicity.

### GzB-IL18 stimulates DCs and polarizes macrophages toward a pro-inflammatory phenotype

Finally, to understand immunological effects of (m)GzB-IL18, we performed phenotyping on splenocytes collected from mice in the experiment described in the preceding paragraph (gating strategy; Figure S9B). We found that the frequency of M1-polarized macrophages was elevated in mice treated with *m2G-T +(m)GzB-IL18* CAR T cells, reaching significance when compared with the *m2G-T +(m)pro-IL18* or *m2G-T + (m)const. IL18* groups (Figure 8D). Total macrophages and M2-polarized macrophages were numerically similar in all groups (Figure S9C). Additionally, DC frequency within the spleens of mice treated with *m2G-T + (m)GzB-IL18* CAR T cells was increased compared to both *m2G-T + (m)pro-IL18* and *m2G-T + (m)const. IL18* groups (where this difference reached significance; Figure 8E). These results indicate that (m)GzB-IL18 can exert a systemic myeloid cell re-programming effect in mice.

## Discussion

Although IL18 enhances the anti-tumor activity of CAR T cells,^4^^,^^5^^,^^14^^,^^27^^,^^41^ this cytokine has been linked to multiple inflammatory disorders in humans.^17^^,^^18^^,^^19^^,^^20^^,^^42^ To mitigate risk of uncontrolled inflammation-related pathology, it is desirable to couple the biological function of IL18 to circumstances in which CAR T cells recognize cognate antigen. Human pro-IL18 is naturally cleaved by caspase 1 on the C-terminal side of aspartic acid 36, thereby releasing the active form of this cytokine. Similar to caspase family members, GzB shares a propensity to cleave peptide substrates adjacent to aspartic acid residues. We found that, by modifying the caspase 1 cleavage site in IL18 to that optimized for human GzB recognition, the resultant GzB-IL18 cytokine remains latent when produced by CAR T cells in the absence of an activating stimulus. Stringency of this system is emphasized by the lack of elevated IFN-γ production by non-activated GzB-IL18-armored CAR T cells. Activation-dependent biological activity of GzB-IL18 in αβ and γδ CAR T cells is indicated by induction of nuclear factor κB (NF-κB)/AP1 activity in IL18-selective reporter cells, accompanied by enhanced tumor cytolytic capacity, IFN-γ release, T cell proliferation, and tumor control in both immunodeficient and immune-competent mice. Although levels of biologically active IL18 achieved using GzB-IL18 were not as high as with constitutively active (const.) IL18, *in vivo* therapeutic efficacy was similar in both cases. Moreover, anti-tumor activity was not further enhanced when GzB-IL18 was co-expressed with additional GzB, arguing that GzB is not limiting for the function of this system. Indeed, some leakiness may have been introduced into the system when GzB was over-expressed together with GzB-IL18, indicated by higher levels of IFN-γ in these cultures. Consequently, co-expression of GzB-IL18 with a CAR of interest represents the optimal application of this technology in our hands.

Caspase-1 activation requires the assembly of cytosolic inflammasomes, which are predominantly found in innate myeloid cell types, although inflammasomes have also been reported in T cells under certain circumstances.^43^ Furthermore, caspase 1 activity has been described in Th17 cells^44^ and human immunodeficiency virus-1-infected CD4^+^ T cells.^45^ In addition, pro-IL18 itself has been reported to be cleaved very slowly by GzB itself.^46^^,^^47^ Nonetheless, we did not detect activation of pro-IL18 following CD3+CD28 crosslinking or tumor cell exposure in any of the CAR T cell populations we studied. In keeping with this, caspase 1 is reported to remain in the inactive pro-enzyme state when T cells are activated.^48^

IL18 is a leaderless protein that is produced in the cytoplasm and released extracellularly via a poorly understood pathway. Physiological IL18 secretion requires at least one and often two additional stimuli.^49^ By contrast, we observed that pro-IL18-armored CAR T cells secrete this cytokine constitutively and in an activation-independent manner. IL18 release has been linked to pyroptosis, which is a form of active cell death in which extensive Gasdermin pore formation occurs.^50^ However, we observed no alteration in the viability or yield of T cells engineered to produce any of the IL18 variants described here, suggesting that release of IL18 is not intrinsically linked to cell death of CAR T cells. Notably, sublethal pore formation by Gasdermin D has been implicated in the release of IL1 family members, including IL18, from living macrophages.^51^ Consequently, the role of Gasdermin proteins and GzB itself (which can cleave Gasdermin family members^52^) in the release of IL18 by these engineered T cells warrants further investigation.

Granzyme B is contained within memory CD8^+^ and CD4^+^ T cells and is upregulated upon activation and by common gamma cytokines. Consequently, the reasons why GzB-IL18 remains latent in non-activated CAR T cells warrant further consideration.^53^ In contrast to IL18, GzB enters the canonical secretory pathway via the endoplasmic reticulum/Golgi apparatus, and thereafter is directed by mannose 6-phosphate tagging to cytolytic granules. At this location, GzB remains enzymatically inactive due to acidic pH and storage on a serglycin scaffold.^28^ When T cells undergo activation, lytic granules fuse with the plasma membrane, releasing GzB extracellularly where it acquires biological activity due to the rapid increase in ambient pH. Given that pro-IL18 (or GzB-IL18) does not enter the canonical secretory pathway, it should only encounter functional GzB extracellularly, following T cell activation and degranulation. The failure of active GzB to encounter GzB-IL18 intracellularly is likely to account for the highly stringent link between biological activity of GzB-IL18 and CAR T cell activation.

Previous studies have confirmed that a major mechanism by which IL18 favors CAR T cell immunity is via autocrine stimulation of the CAR T cells themselves.^5^^,^^14^^,^^41^ However, IL18 also exerts several paracrine effects on the TME, as described in the section “introduction.” Moreover, broadened immune reactivity through epitope spreading accompanied by enhanced macrophage-dependent anti-tumor activity has been observed.^14^ To characterize the ability of GzB-IL18 to harness similar mechanisms, studies using immune-competent mouse models are necessary. Furthermore, human IL18 has unproven biological activity in the mouse, mandating the evaluation of murine variants of this cytokine for both immunomodulatory effects and safety *in vivo*.

To address these questions, we modified the caspase 1 cleavage site in mouse IL18 to one preferred by murine granzyme B.^38^ This cytokine was co-expressed with a mouse CAR with specificity for the panErbB network (*m2G-T*) and with the capacity to trigger dose-dependent CRS.^39^^,^^54^ When armored with constitutively active IL18, doses of as little as 4 million CAR T cells induced lethal CRS in Balb/c mice. Although IL18 has shown a favorable safety profile in patients when expressed in CD19 CAR T cells,^55^ the restricted expression pattern of this target may underestimate the potential for IL18-induced toxicity. By contrast, widespread expression of ErbB dimers in normal tissues is very likely to have been a key driver of the toxicity we observed with (m)const. IL18-armored CAR T cells. Importantly, however, such toxicity was not evident when *m2G-T* CAR T cells were armored with (m)GzB-IL18, demonstrating the superior safety profile of this variant. Furthermore, these T cells exhibited greater therapeutic activity in mice engrafted with an s.c. HNSCC tumor when compared to unarmored CAR T cells or those that co-expressed (m)pro-IL18. We also found that (m)GzB-IL18 induced systemic myeloid cell re-programming in the mice, indicated by an increase in splenic M1-polarized macrophages and a trend toward elevated splenic DC number. This contrasts with the lack of such systemic effects in response to (m)const. IL18, a finding that was also reported recently by Brentjens et al., who found that such alterations occurred selectively within the TME.^56^ This surprising result raises the possibility that myeloid re-programming effects of IL18 can be separated from its CRS-promoting activity, perhaps because of differing intensity and/or kinetics of biological activity of GzB-IL18 when compared to the constitutively active form of this cytokine. Unfortunately, we were unable to analyze the TME since excised tumors were too small for successful retrieval of myeloid cells. Similarly, we were unable to analyze mice for evidence of epitope spreading in light of the difficulty in generating an entirely ErbB-negative version of this tumor cell line.

Armoring with IL18 exerted significant metabolic influences upon the host CAR T cells, but these were largely confined to the γδ rather than αβ subset. Upregulated expression of nutrient transporters in these cells recapitulates the known effect of IL18 on NK cells.^57^ IL18 also promotes glycolysis in NK cells.^57^ In keeping with this, we observed increased GLUT1 expression by CAR γδ T cells and a non-significant shift to glucose-dependent metabolism under conditions of tumor re-stimulation, which might underpin the increased effector function. In addition, we also observed that mitochondrial mass was increased and mitochondrial metabolism maintained in IL18-armored γδ CAR T cells, consistent with a metabolically fitter phenotype overall. Collectively, these findings may have contributed significantly to the strong armoring effect of IL18 in CAR γδ T cells, favoring tumor eradication in most cases where CAR γδ T cells alone achieved much poorer disease control.

One potential concern is the risk of leakiness of the system when co-expressed with a CAR that displays a high level of tonic signaling.^58^ Accordingly, toxicity may be dependent on intrinsic properties of the CAR. However, it is generally undesirable to undertake clinical development of CARs with a high propensity to tonic signaling.

In conclusion, we describe a simple strategy to harness the armoring effects of IL18, while mitigating risks associated with constitutive release of this cytokine. Since the GzB-IL18 transgene is of modest size, we envision that this system may find application in many experimental CAR T cell immunotherapy approaches for cancer.

## Materials and methods

### Retroviral constructs

The SFG retroviral vector was used to express all transgenes. All synthetic cDNAs were generated by Genscript (Leiden, the Netherlands). The SFG vector used to co-express firefly luciferase and tdTomato red fluorescence protein (SFG ffLuc Tom) in tumor cell lines has been described.^35^ The *pCAR-H/T* parallel CAR construct has also been described and is referred to here as *pCAR-H/T + nil*.^31^ To generate *pCAR-H/T + pro-IL18*, a codon-optimized cDNA was synthesized to encode CD3ζ (codon 92 - end; stop codon removed, incorporating a proximal KflI restriction site), furin cleavage site (RRKR), *Thosea Asigna* 2A ribosomal skip peptide (codon wobbled with respect to that in SFG *pCAR-H/T*), human pro-IL18, stop codon, and XhoI restriction site. This fragment was digested using KflI and XhoI and ligated with the unique KflI and XhoI restriction sites in SFG *pCAR-H/T*, replacing the 224-bp fragment that is removed. SFG *pCAR-H/T + GzB-IL18* was generated by site-directed mutagenesis of SFG *pCAR-H/T + pro-IL18*, modifying GAC GAC GAG AAC CTG GAG AGC GAC TAC to GAC GAC GAG AAC ATC GAG CCC GAC TAC. To generate SFG *pCAR-H/T + const. IL18*, a codon-optimized cDNA was synthesized to encode CD3ζ (codon 92 - end; stop codon removed, incorporating a proximal KflI restriction site), furin cleavage site (RRKR), *Thosea Asigna* (T2A) ribosomal skip peptide (codon wobbled with respect to that in SFG *pCAR-H/T*), CD4 leader peptide fused to mature human IL18 (codons 37–193), stop codon, and XhoI restriction site. This fragment was digested using KflI and XhoI and ligated with the unique KflI and XhoI restriction sites in SFG *pCAR-H/T* as above. The SFG *pCAR-H/T* + *GzB-IL18/GzB* vector was generated in two steps. First, a GzB cDNA was inserted into SFG-*pCAR-H/T*. A codon-optimized cDNA was synthesized to encode CD3ζ (codon 92 - end; stop codon removed, incorporating a proximal KflI restriction site), furin cleavage site (RRKR), *Porcine Teschovirus-1* (P2A) ribosomal skip peptide, human GzB, stop codon, and XhoI restriction site. This fragment was digested using KflI and XhoI and ligated with the unique KflI and XhoI restriction sites in SFG *pCAR-H/T* as above to give SFG-*pCAR-H/T + GzB*. Next, a codon-optimized cDNA was synthesized to encode GzB (codon 231 - end; stop codon removed, incorporating a proximal AleI restriction site), furin cleavage site (RRKR), T2A ribosomal skip peptide (codon wobbled with respect to that in SFG *pCAR-H/T*), GzB-IL18, stop codon, and XhoI restriction site. This fragment was digested using AleI and XhoI and ligated with the unique AleI and XhoI restriction sites in SFG *pCAR-H/T*, replacing the 55-bp fragment that is removed.

The SFG 2G-T (CD28) CAR (here referred to as *2G-T*) has been previously described.^31^ A similar cloning strategy to that described above was used to generate *2G-T + GzB-IL18*.

To generate the murine pan-ErbB-specific *m2G-T* CAR, a codon-optimized cDNA was synthesized to encode murine T1E peptide (placed downstream of a CD8α leader peptide), mouse CD28 hinge, and transmembrane domain (amino acids 115–177) followed by the intracellular domain of mouse CD3ζ and a stop codon. This fragment was flanked with a 5′ Nco1 site (incorporating the start codon) and 3′ Xho1 site allowing ligation into the SFG vector following digestion with these enzymes. To produce each murine IL18-armored variant, SFG *m2G-T* was digested with Pmi1 and Nco1 and a fragment inserted encoding a short sequence from the vector backbone followed by mouse pro-IL18 (Uniprot: P70380), furin cleavage site (RRKR), and *Thosea Asigna* 2A ribosomal skip peptide, thereby giving SFG *m2G-T + (m)pro-IL18* in which (m)pro-IL18 lies upstream of *m2G-T*. The (m)pro-IL18 sequence was converted to (m)GzB-IL18 by mutagenesis of the amino acid sequence LESD to IEFD (Figure 8A), giving SFG *m2G-T + (m)GzB-IL18*. To engineer (m)const. mIL18, a cDNA fragment was synthesized encoding for vector backbone, mouse CD4 leader fused to amino acids 36–192 of mouse IL18, RRKR, and *Thosea Asigna* 2A ribosomal skip peptide was inserted into the Pmi1 and Nco1 sites in SFG *m2G-T* as described above, giving SFG *m2G-T + (m)const. IL18*.

### Culture and retroviral transduction of primary human T cells

Blood samples were obtained from healthy male and female volunteers aged between 18 and 65 years with approval of a National Health Service Research Ethics Committee (reference 18/WS/0047). Activation of unfractionated T cells was achieved 48 h prior to gene transfer using phytohemagglutinin 5 μg/mL; Merck, Darmstadt, Germany) or CD3+CD28-coated paramagnetic beads (1:1 bead:cell ratio; Thermo Fisher Scientific, Paisley, UK). Retroviral vector was prepared by triple transfection of 293T cells and transduction of activated T cells was conducted in RetroNectin-coated non-tissue-culture-treated plates, all as described.^31^ Transduced T cells were cultured in RPMI-1640 supplemented with 5% human AB serum (Merck), GlutaMax, and antibiotic-antimycotic solution (Thermo Fisher Scientific) and in the presence of 100 U/mL IL2 (Proleukin, Clinigen, London, UK).

Activation of γδ T cells was achieved using immobilized pan-γδ T cell receptor (TCR) antibody (0.8 μg/mL 11F2 clone; BD Biosciences, San Jose, CA). Thereafter, γδ T cells were expanded in the presence of IL2 (100 U/mL) and TGF-β (5 ng/mL, Bio-Techne, Abingdon, UK). Retroviral transduction of activated γδ T cell cultures was carried out 72 h after activation as described above except that retrovirus-containing supernatant (1 mL) was pre-immobilized on a RetroNectin-coated non-tissue-culture-treated 24-well plate at 4°C overnight. After removal of the vector, activated γδ T cells (total of 0.5 million activated peripheral blood mononuclear cells [PBMCs]) were added to the well together with IL2 (100 U/mL).

### Culture and retroviral transduction of primary mouse T cells

Retroviral vector for transduction of mouse T cells was prepared by plating of 1.65 × 10^6^ Phoenix Eco cells (American Tissue Culture Collection, Manassas, VA) per 10-cm dish in D10 medium, comprising DMEM (Lonza, Basel, Switzerland) supplemented with 10% FBS (Merck), GlutaMax, and antibiotic-antimycotic solution (both from Thermo Fisher Scientific). After 24 h, cells were transfected with 4.7 μg of Peq-Pam 3 (gift of Dr. M. Pule, University College London, UK) and 4.7 μg of retroviral plasmid using GeneJuice (Merck). Retroviral supernatant was harvested at 48 and 72 h post transfection and used fresh. Spleens were harvested from 8-week-old BALB/c mice followed by magnetic-activated cell sorting of untouched T cells (130-095-130, Miltenyi Biotec, Bergisch Gladbach, Germany). T cells were activated at a 1:1 ratio with CD3+CD28-coated paramagnetic beads (11456D, Thermo Fisher Scientific) and cultured in D10 medium, supplemented with 100 IU/mL IL2. Transduction of activated T cells was conducted at 24 h post activation in a RetroNectin-coated non-tissue-culture-treated plate as described in the preceding section. T cells were expanded for 7 days post activation before infusion into BALB/c mice.

### Cell lines

MDA-MB-468 TNBC cells were obtained from the Breast Cancer Now Research Unit, King’s College London, UK. MDA-MB-435 cells were a gift of Professor Joy Burchell, King’s College London. BxPC3 cells were a gift of Professor John Marshall, Barts Cancer Institute, Queen Mary University of London, UK. The B7E3 murine HNSCC tumor cell line was a gift of Dr C van Waes (National Institute on Deafness and Other Communication Disorders, Bethesda, MD). Where indicated, sub-confluent tumor cell monolayers were repeatedly transduced with SFG ffLuc Tom supernatant derived from a stable PG13 retroviral packaging cell line, following passage through a 0.44-μM filter (Sartorius Stedim, Göttingen, Germany). Tumor cell lines were grown in D10 medium. All tumor cell lines were validated by short tandem repeat DNA profiling and subjected to regular mycoplasma screening. All experiments were performed within 30 passages.

### Flow cytometry analysis

Prior to all analyses, cells were washed with 1× PBS. Antibody incubations were conducted in 1× PBS (unless otherwise stated) for 30 min at 4°C protected from light. The following fluorophore-conjugated antibodies were used for staining: CD98-phycoerythrin cyanine 7 (PE-Cy7; BioLegend, San Diego, CA), γδ-TCR-PE (Immunotech, Beckman Coulter, Brea, CA), or fluorescein isothiocyanate (FITC)-conjugated pan-γδ TCR antibody (IMMU510, Beckman Coulter, High Wycombe, UK). Cells were stained concurrently with 2 μL/mL fixable Live/Dead dye (Thermo Fisher Scientific). To detect expression of *pCAR-H/T* + nil and armored IL18 derivatives, cells were incubated with 1 μg of MUC1 60mer-biotin (VTSAPDTRPAPGSTAPPAHG)3 (NeoMPS) followed by streptavidin-APC (allophycocyanin) or -PE conjugate (BioLegend), which detects CAR expression. Expression of the *pCAR-H/T* CCR and *2G-T* CAR were detected using anti-human epidermal growth factor (EGF) (10825; R&D Systems, Minneapolis, MN) followed by streptavidin-APC or -PE conjugate. To detect GLUT1, cells were fixed with FOXP3 fix/perm kit (Thermo Fisher Scientific) for 30 min protected from the light. Cells were stained with GLUT1-APC antibody (Abcam, Cambridge, UK) in permeabilization buffer (FOXP3 fix/perm kit, Thermo Fisher Scientific). To stain mitochondria, cells were incubated with 50 nM Mitotracker Deep Red FM and 5 nM Mitotracker Green FM (Thermo Fisher Scientific) in RPMI for 20 min at 37°C, 5% CO_2_. Cells were washed with ice-cold PBS and stained for surface markers (CD3-APC-Cy7, γδ-TCR-PE [where relevant] and Live/Dead dye) as previously described prior to flow cytometry analysis.

Expression of the *m2G-T* CAR was detected using anti-mouse EGF (500-P174GBT, PeproTech EC) followed by streptavidin-PE conjugate (BioLegend).

To analyze mouse splenocytes, spleens were harvested and mashed through a 0.7-μm cell strainer. Cells were pelleted and resuspended in PBS before staining with the following anti-mouse antibodies: CD3-BV785, B220-BV785, NK1.1-BV785 (lineage markers), CD45-BV510, CD11b-BV421, F4/80-PEcy/7, Gr-1-PE, CD11c-BV605, MHC-CII-FITC (all from BioLegend). Macrophages were defined as lineage marker negative (Lin^−^), F4/80^+^, and CD11b^+^. M1-polarized macrophages were identified as Lin^−^, CD11b^+^, F4/80^+^, Gr-1^−^, and MHC-II^+^. M2-polarized macrophages were defined as Lin^−^, CD11b^+^, F4/80^+^, Gr-1^−^, and MHC-II^−^. DCs were identified as CD11b pos, CD11c pos, MHC-II pos.

Flow cytometry was performed using a Becton Dickenson Fortessa or Beckman Coulter Cytoflex cytometer with FACSDiva or FlowJo software.

### ELISA

Supernatants from tumor/T cell co-cultures were analyzed using a human IFN-γ (Thermo Fisher Scientific, Waltham, MA), human IL18, human TNF-⍺, or human IL2 ELISA kit (all R&D systems), as described by the manufacturers. Mouse IL18 was measured in supernatants collected from transduced mouse T cells by ELISA (R&D systems).

### HEK-Blue IL18 reporter cell assay

1 × 10^5^ T cells were co-cultured with 1 × 10^4^ tumor cells, anti-CD3/28 TransAct beads (as recommended by the manufacturers; Miltenyi Biotec), or media for 24 h in R5 medium. HEK-Blue IL18 reporter cells (InvivoGen, Toulouse, France) were cultured for 24 h in D10 medium and then stimulated with supernatant from co-cultures for 24 h. Supernatant from stimulated HEK-Blue IL18 reporter cells was developed according to manufacturer’s instructions.

### Cytokine array

Cytokines were measured in the sera of BALB/c mice using the LEGENDplex Mouse Inflammation panel (13-plex) (BioLegend) following the manufacturers’ instructions.

### Tumor cell cytotoxicity assays

Tumor cell monolayers (96-well plate) were incubated with T cells for 24–72 h at specified effector to target (E:T) ratios. Destruction of tumor cell monolayers by T cells was quantified using an MTT (3-[4,5-dimethylthiazol-2-yl]-2,5 diphenyl tetrazolium bromide) or luciferase assay. In the former, T cells were removed and MTT (Merck) was added at 500 μg/mL in fresh D10 medium for 1–2 h at 37°C and 5% CO_2_. After removal of the supernatant, formazan crystals were resuspended in 50 μL of DMSO. Absorbance was measured at 560 nm. In luciferase assays, D-luciferin (PerkinElmer, Waltham, MA) was added at 150 μg/mL immediately prior to luminescence reading. Tumor cell viability was calculated as (absorbance or luminescence of monolayer cultured with T cells/absorbance or luminescence of untreated monolayer alone) × 100%.

### Tumor re-stimulation assays

CAR T cells were added to MDA-MB-468 or BxPC3 tumor cells at a 1:1 E:T ratio (1 × 10^4^ tumor cells). Tumor viability was determined after 72 h by MTT or luciferase assay and T cells were transferred to a fresh well containing 1 × 10^4^ tumor cells. T cells were re-stimulated in this manner until they could no longer be retrieved from tumor monolayers. A stimulation cycle was deemed successful if ≥60% of tumor cells were destroyed. Supernatant was harvested after 72 h for measurement of IFN-γ by ELISA.

### Metabolic flux analysis (SCENITH)

Methods were adapted from those described by Arguello et al.^59^ For each sample, T cells (1 million/mL) were split into five wells in a 96-well plate and incubated at 37°C for 45 min with puromycin (20 μg/mL) in combination with either 1× PBS (PBS control), 2-deoxy-D-glucose (2DG; 100 mM), Oligomycin-A (O; 1 μM), a combination of 2DG + O or Harringtonine (negative control). Following incubation, T cells were washed in ice-cold PBS and then stained for surface markers (CD3-APC-Cy7, γδ-TCR-PE, Live/Dead dye) as previously described. Puromycin was stained intracellularly using the FOXP3 fix/perm kit (Thermo Fisher Scientific) as described in the “flow cytometry analysis” section using AF488-puromycin antibody (Merck). The following calculations were used to determine glucose and oxygen dependence.^59^

$$1.\;Glucose\;Dependence\;\left(\%\right)=\left(\frac{Puro\;{GMFI}_{PBS\;control}-Puro\;{GMFI}_{2DG}}{{Puro\;GMFI}_{PBS\;control}-{Puro\;GMFI}_{2DG+Oligomycin}}\right)\times 100$$

$$2.\;Oxygen\;Dependence\;\left(\%\right)=\left(\frac{Puro\;{GMFI}_{PBS\;control}-Puro\;{GMFI}_{oligomycin}}{{Puro\;GMFI}_{PBS\;control}-{Puro\;GMFI}_{2DG+Oligomycin}}\right)\times 100$$

### *In vivo* studies

All *in vivo* experimentation adhered to UK Home Office guidelines, as specified in project licence number 70/7794 or P23115EBF and was approved by the King’s College London animal welfare and ethical review body (AWERB). Female SCID Beige mice (Charles River Laboratories, Alderley Park, UK) were 6–10 weeks old when used for experiments. MDA-MB-468 cells that had been transduced with SFG ffLuc Tom were inoculated using the intraperitoneal route (1 × 10^6^ tumor cells per mouse). BLI was performed using an IVIS Spectrum Imaging platform (PerkinElmer) with Living Image software (PerkinElmer). To image tumor status, mice were injected i.p. with D-luciferin (150 mg/kg; PerkinElmer) and imaged under isoflurane anesthesia after 20 min. Image acquisition was conducted on a 15- or 25-cm field of view with medium binning and auto-exposure. Mice were allocated to experimental groups based on similar average tumor burden prior to treatment, which was administered on day 11. Treatment consisted of 10 × 10^6^ engineered αβ or γδ T cells as indicated in individual experiments.

Immunocompetent studies were performed in female 6- to 10-week-old BALB/c mice (Charles River Laboratories). B7E3 (1 × 10^5^ tumor cells per mouse) were inoculated using the s.c. route and tumor engraftment was monitored by caliper measurements. Mice were allocated to experimental groups based on similar average tumor burden. On day 13, animals received cyclophosphamide 50 mg/kg i.p. followed on day 14 by i.v. infusion of either 2 × 10^6^ or 4 × 10^6^ engineered BALB/c T cells as indicated in individual experiments. In all experiments, animals were inspected daily and weighed at the specified intervals. Mice were culled if symptomatic due to tumor progression, elevated toxicity score (measured exactly as described),^40^ or weight loss of ≥15% (except that a 20% threshold applied if weight loss was attributed to CRS).

### Statistical analysis

All data are derived from biological replicates involving independent donors unless otherwise indicated. For analysis of multiple groups, statistical analysis was performed using one-way or two-way ANOVA test (depending on the number of independent variables) followed by Tukey’s multiple-comparisons test. Survival data were analyzed using a log rank (Mantel-Cox) test. When only two groups were compared, an unpaired Student’s t test was performed. One sample t test was used to compare the mean of an experimental group to that of a normalized control population. All statistical analyses were performed using GraphPad Prism version 9.1.

## Data and code availability

The data generated in this study are presented within the article and supplemental information files. Other data are available upon request.

## Supplemental information

Document S1. Figures S1–S9

Document S2. Article plus supplemental information
