# Supplementary material for: Granzyme B-activated IL18 potentiates αβ and γδ CAR T cell immunotherapy in a tumor-dependent manner
Source: Mol Ther. 2024 May 14;32(7):2373–92. doi: 10.1016/j.ymthe.2024.05.013 (PMC11286818; doi:10.1016/j.ymthe.2024.05.013)
Supplement: Document S1. Figures S1–S9 [file mmc1.pdf]

YMTHE, Volume *32*

Supplemental Information

Granzyme B-activated IL18 potentiates $\alpha\beta$ and $\gamma\delta$ CAR T cell immunotherapy in a tumor-dependent manner

Caroline M. Hull, Daniel Larcombe-Young, Roberta Mazza, Molly George, David M. Davies, Anna Schurich, and John Maher

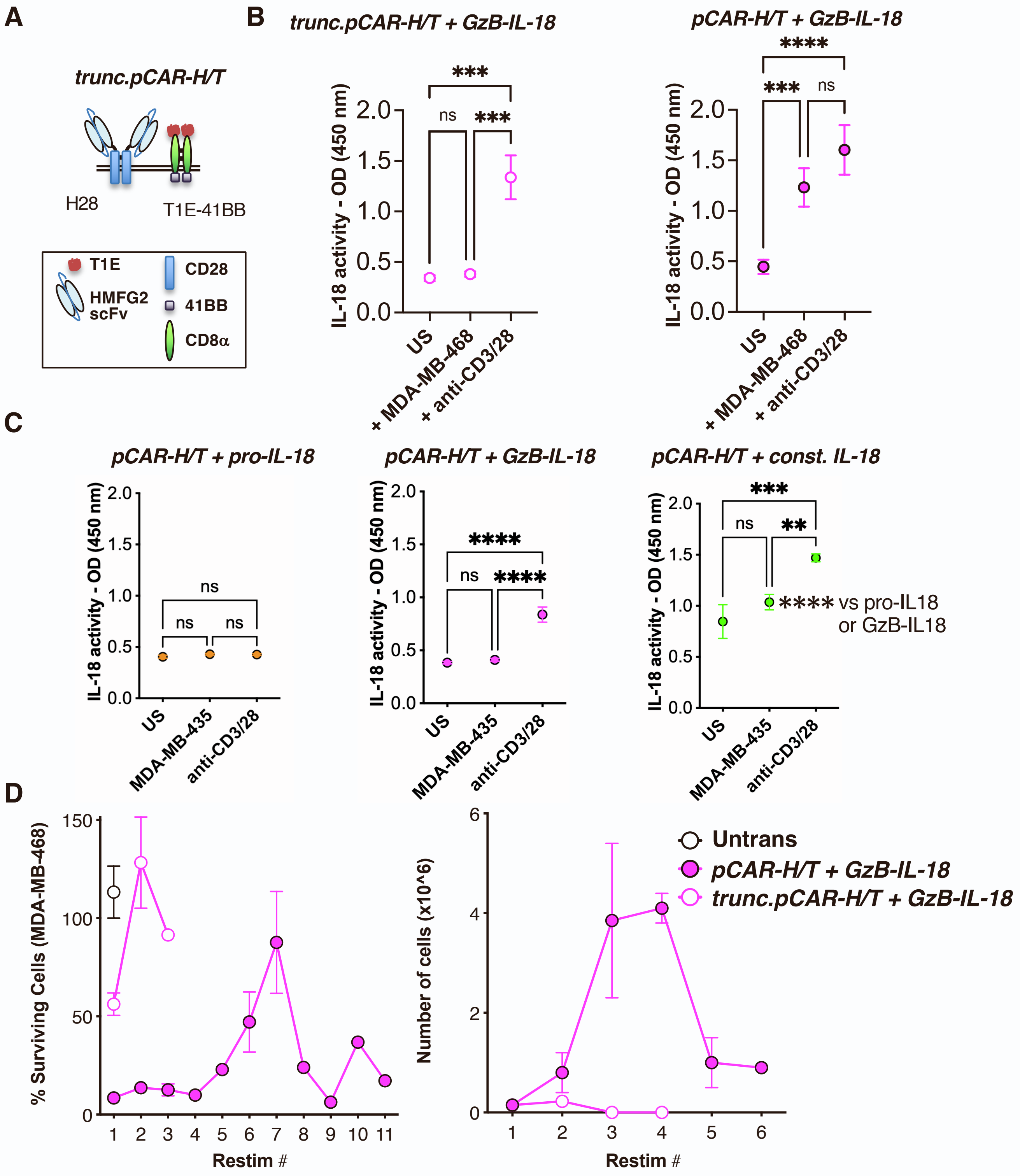

Figure S1: CAR dependent induction of IL-18 activity in pCAR T-cells co-expressing GzB-IL18.
(A) The *pCAR-T/H + GzB-IL18* construct was altered to remove the CD3ζ endodomain from the CAR, thereby generating *trunc.pCAR-T/H + GzB-IL18.* **(B)** T-cells were transduced with the indicated retroviral vectors then plated at a density of 5 x 10^5 cells/mL alone (US – unstimulated), or were co-cultured with MDA-MB-468 cells (at a ratio of 5 to 1) or anti-CD3/CD28 TransAct™ beads. Supernatants were collected after 48 hours and analyzed for IL18 biological activity using HEK-Blue™ IL-18 reporter cells (mean +/- SEM, n=4 donors measured in triplicate). ****$p < 0.0001$, ***$p < 0.001$, ns – not significant using two-way ANOVA. (**C**) Assessment of functional activity of IL18 produced by the indicated IL18 armored CAR T-cells when co-cultured with MUC1 negative MDA-MB-435 cells (mean +/- SEM, n=6). ****$p < 0.0001$, ***$p < 0.001$, **$p < 0.01$ ns – not significant using two-way ANOVA. **(D)** CAR T-cells (or untransduced T-cells as control) were added to MDA-MB-468 tumor cells at a 1:1 effector to target ratio (1 x 10^4 tumor cells). Tumor viability was determined after 72 hours (left; mean +/- SEM, n=9-12) and T-cells were transferred to a fresh well containing 1 x 10^4 tumor cells. T-cells were re-stimulated (restim) in this manner until ≤60% of tumor cells were destroyed. Cells were counted at the end of each restimulation (right; mean +/- SEM, n=2).

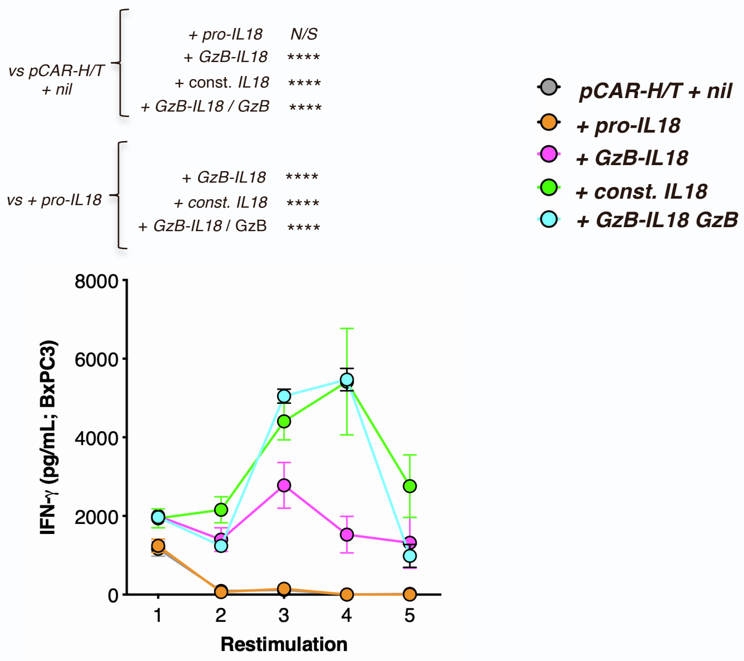

Figure S2: Cytokine production by CAR T-cells during co-culture with tumor cells.
(A) CAR T-cells were added to BxPC3 tumor cells at a 1:1 E:T ratio (1 x 10^4 tumor cells). Supernatant was collected after 72 hours for IFN-γ ELISA and T-cells were transferred to a fresh well containing 1 x 10^4 tumor cells. T-cells were re-stimulated and supernatant collected in this manner until $\leq$60% of tumor cells were destroyed. Serial IFN-γ production is shown (mean +/- SEM; n = 6-15). ****$p < 0.0001$ by two-way ANOVA.

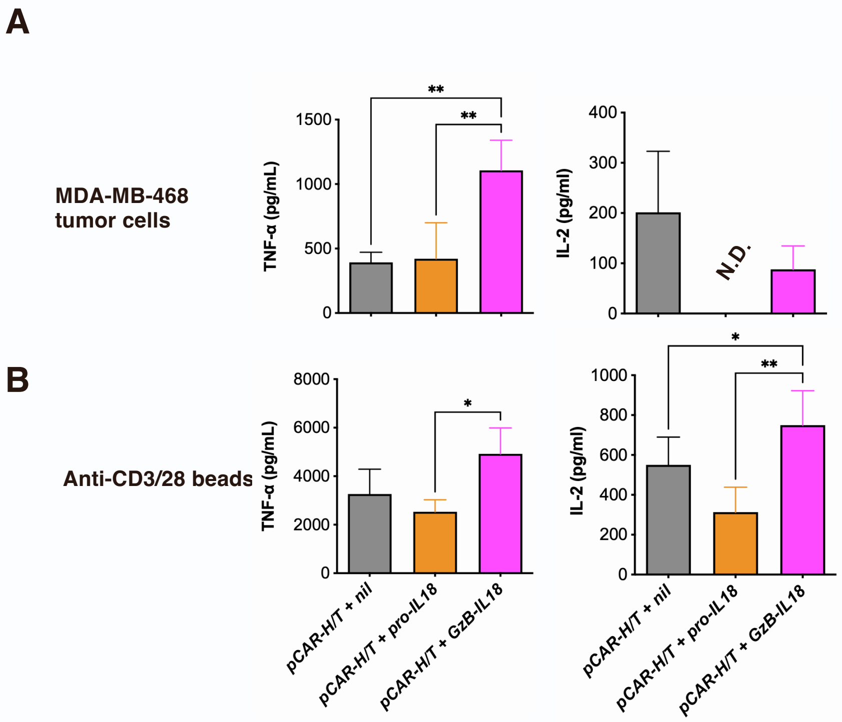

Figure S3: Cytokine production by IL18-armored CAR T-cells during co-culture with tumor cells.

CAR T-cells were plated at a density of 5 x 10^5 cells/mL and co-cultured with MDA-MB-468 cells (at a ratio of 5 to 1) or anti-CD3/CD28 beads. Supernatants were collected after 48 hours and analyzed for TNF-α (**A**) and IL-2 (**B**) by ELISA (mean +/- SEM, n=1-3 donors in triplicate). $^{**}p < 0.01$, $^{*}p < 0.05$ using two-way ANOVA. (N.D. – not detected).

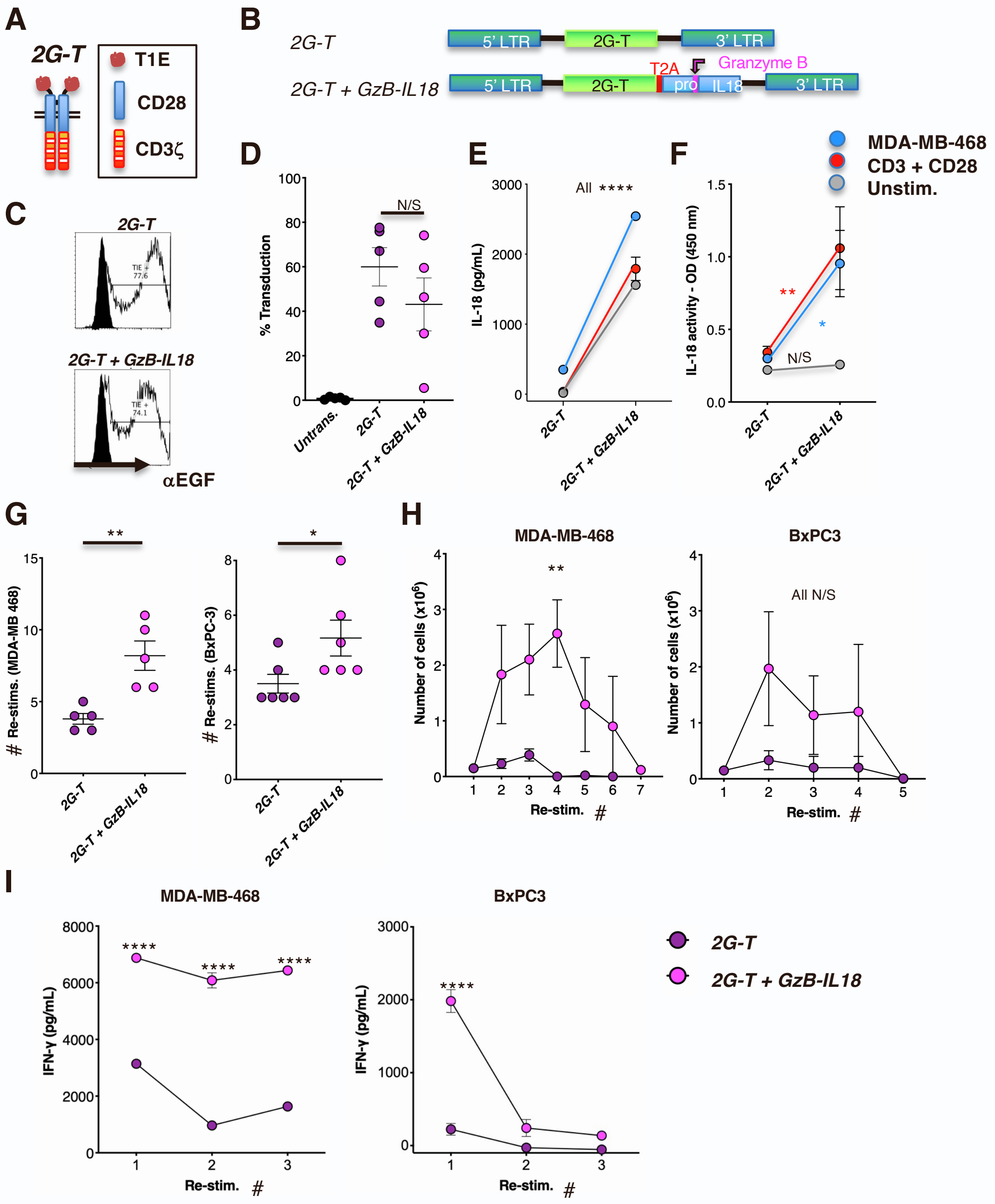

Figure S4: GzB-IL18 promotes ErbB-specific CAR T-cell function *in vitro*.
(**A**) Structure of the *2G-T* second generation CAR, with specificity for 8 of 9 ErbB homo- and heterodimers. (**B**) Structure of SFG retroviral vector that encodes *2G-T*, either alone, or in combination with GzB-IL18. (**C**) Representative histograms to illustrate cell surface expression of *2G-T* on day 11 after T-cell transduction with the SFG vectors shown in panel B. (**D**) T-cell transduction efficiency with SFG *2G-T* and GzB-IL18 armored derivative on day 11 (mean +/- SEM, n = 4). N/S using one-way ANOVA. (**E**) T-cells were transduced with the indicated retroviral vectors, or untrans.(duced) as control. T-cells were then plated at a density of 5 x 10^5 cells/mL alone, or were co-cultured with MDA-MB-468 cells (at a ratio of 10 to 1) or anti-CD3/CD28 beads. Supernatants were collected after 24 hours and analyzed for IL18 by ELISA (mean +/- SEM, n=3). ****$p < 0.0001$ using two-way ANOVA. (**F**) Supernatants described in E were added to HEK-Blue™ IL18 reporter cells to assess IL18 biological activity, measured as optical density (OD) at 450nm (mean +/- SEM, n = 6). **$p < 0.01$, *$p < 0.05$, N/S – not significant by two-way ANOVA. (**G**) CAR T-cells were added to MDA-MB-468 (left) or BxPC3 tumor cells (right) at a 1:1 E:T ratio (1 x 10^4 tumor cells). Tumor viability was determined after 72 hours and T-cells were transferred to a fresh well containing 1 x 10^4 tumor cells. T-cells were re-stimulated in this manner until they could no longer be retrieved from tumor monolayers. A stimulation cycle was deemed successful if $\geq$60% of tumor cells were destroyed. The number of successful re-stimulation cycles for each T-cell/ tumor cell condition is shown (mean +/- SEM). Statistical analysis was by paired Student *t*-test; **$p < 0.01$, *$p < 0.05$. (**H**) T-cell number was determined prior to each tumor re-stimulation cycle (mean +/- SEM, n = 3-7). **$p < 0.01$ by two-way ANOVA. (**I**) IFN-γ was measured in supernatants collected 72 hours after initiation of the first 3 tumor re-stimulation cycles. ****$p < 0.0001$ by two-way ANOVA.

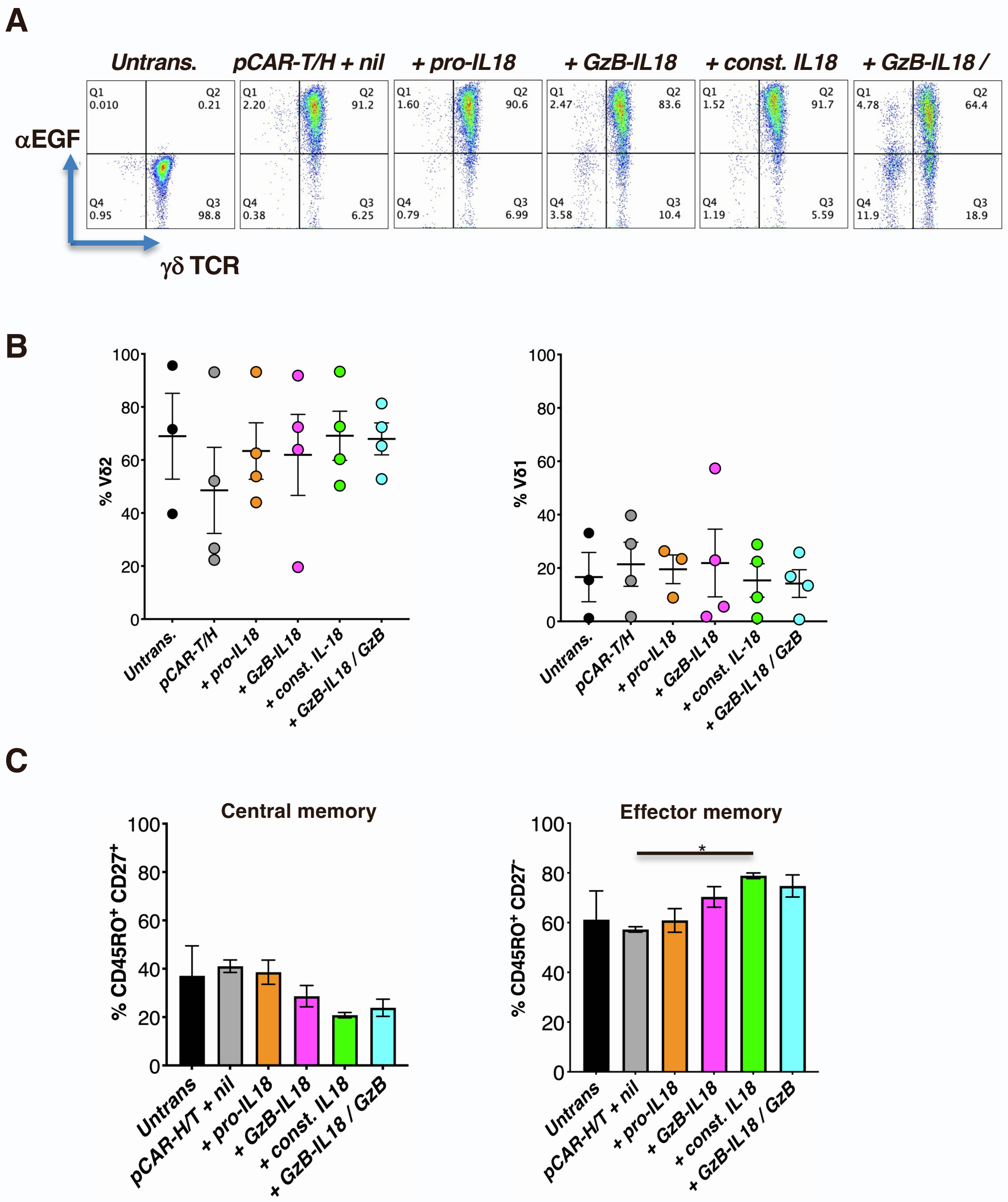

Figure S5. Expansion and phenotype of IL18-armored *pCAR-H/T* γδ T cells.
(**A**) PBMC were activated using an anti-γδ TCR antibody, transduced after 72 hours with the indicated vectors and expanded thereafter in TGF-β + IL-2. Cells were analyzed on day 21 by flow cytometry. γδ TCR expression was determined using a pan-γδ TCR antibody. Expression of *pCAR-H/T* was detected using anti-EGF antibody, which binds to the CCR. (**B**) On day 21 post activation, the proportion of Vδ2 and Vδ1 T-cells present in the indicated cultures was determined by flow cytometry (mean +/- SEM). (**C**) Differentiation status of the cells was also analyzed on day 21. Percentage central memory (CD45RO$^+$ CD27$^+$) and effector memory (CD45RO$^+$ CD27$^-$) cells are indicated (mean +/- SEM, n = 3-4). *$p < 0.05$ by one-way ANOVA.

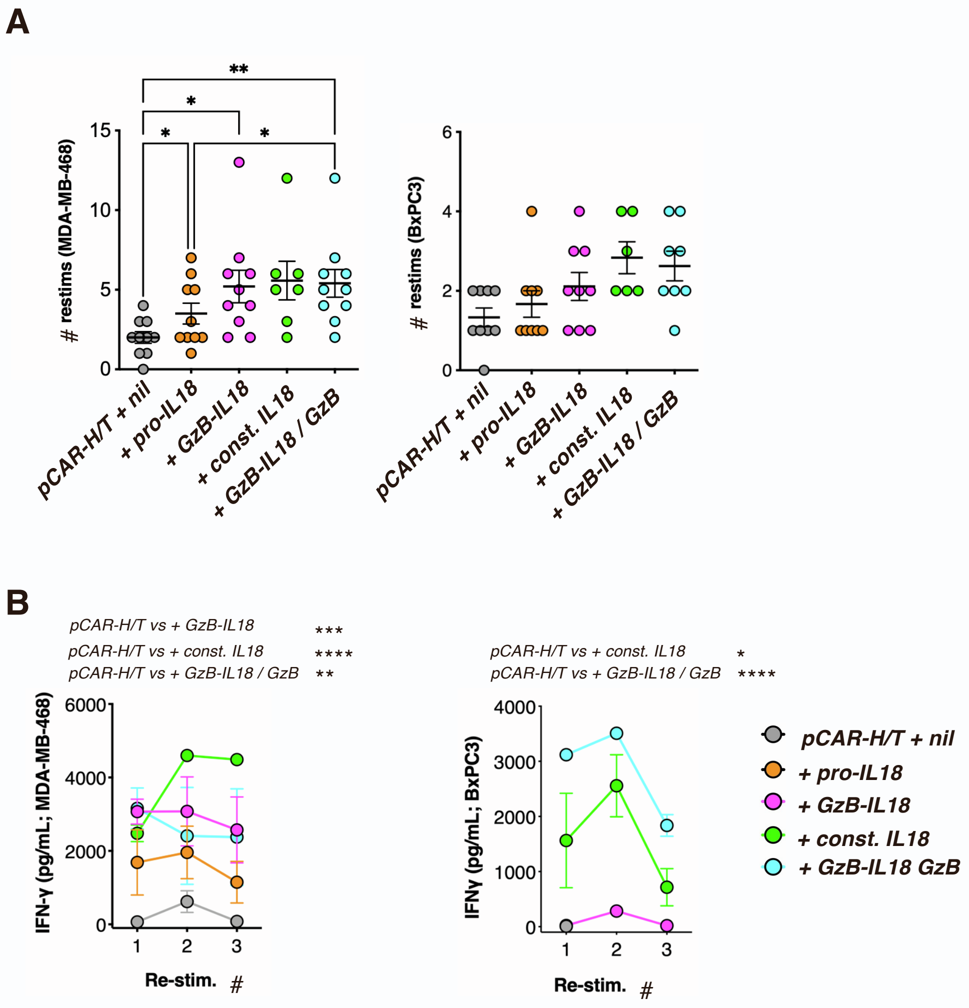

Figure S6. ***In vitro*** **anti-tumor activity of IL18-armored** ***pCAR-H/T*** **γδ T-cells**.

(**A**) γδ T-cells were engineered to express *pCAR-H/T*, either alone or together with the indicated IL18 variant. Transduced cells were added to MDA-MB-468 (left) or BxPC3 tumor cells (right) at a 1:1 E:T ratio (1 x 10^4 tumor cells). Tumor viability was determined after 72 hours and T-cells were transferred to a fresh well containing 1 x 10^4 tumor cells. T-cells were re-stimulated in this manner until they could no longer be retrieved from tumor monolayers. Number of successful re-stimulation cycles is shown (mean +/- SEM). A stimulation cycle was deemed successful if $\geq$60% of tumor cells were destroyed. **$p < 0.01$, *$p < 0.05$ by one-way ANOVA. (**B**) IFN-γ was measured in supernatants collected 72 hours after initiation of the first 3 tumor re-stimulation cycles (mean +/- SEM; n=2-6). ****$p < 0.0001$, ***$p < 0.001$, **$p < 0.01$, *$p < 0.05$ by two-way ANOVA.

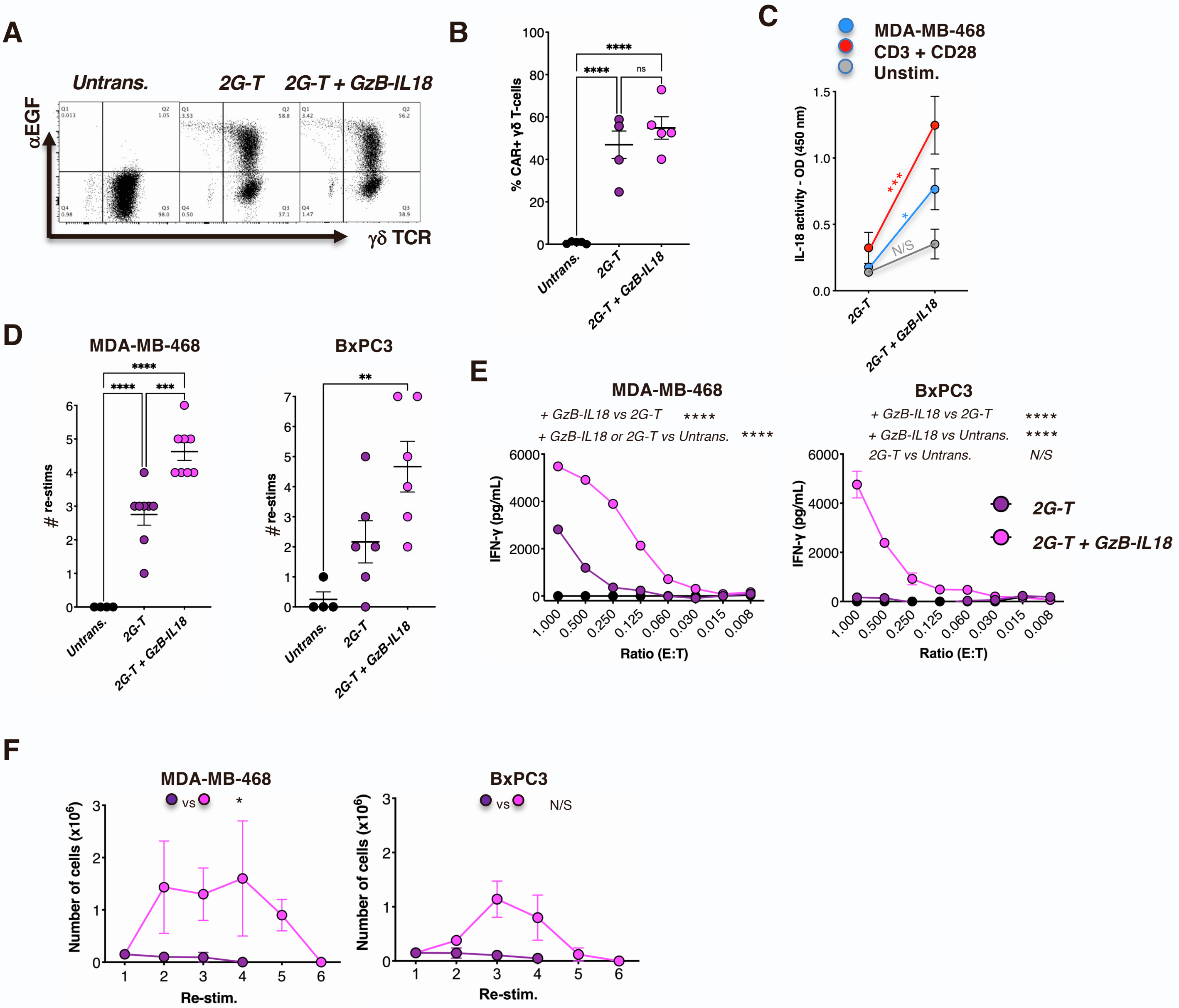

Figure S7. GzB-IL18 promotes *2G-T* CAR γδ T-cell function *in vitro*.
(**A**) Representative examples of 2G-T CAR expression by γδ T-cells transduced with the indicated retroviral vectors. (**B**) Transduction efficiency of replicate donors as determined by flow cytometric analysis of surface CAR expression (mean +/- SEM). ****p < 0.0001, ns – not significant by one-way ANOVA. (**C**) T-cells were transduced with the indicated retroviral vectors, or untransduced as control. T-cells were then plated at a density of 10^5 cells/mL and cultured alone, or were co-cultured with MDA-MB-468 cells (at a ratio of 10 to 1) or anti-CD3/CD28 beads. Supernatants were collected after 24 hours and added to HEK-Blue™ IL18 reporter cells to assess IL18 biological activity, measured as optical density (OD) at 450nm (mean +/- SEM, n = 6). ***p < 0.001, *p < 0.05, N/S not significant by two-way ANOVA. (**D**) γδ T-cells were engineered to express *2G-T*, either alone or *+GzB-IL18*. Transduced cells were added to MDA-MB-468 (left) or BxPC3 tumor cells (right) at a 1:1 E:T ratio (1 x 10^4 tumor cells), making comparison with untransduced control cells. Tumor viability was determined after 72 hours and T-cells were transferred to a fresh well containing 1 x 10^4 tumor cells. T-cells were re-stimulated in this manner until they could no longer be retrieved from tumor monolayers. A stimulation cycle was deemed successful if $\geq$60% of tumor cells were destroyed. ****p < 0.0001, ***p < 0.001, **p < 0.01 by one-way ANOVA. (**E**) IFN-γ was measured in supernatants collected 72 hours after initiation of the first 3 tumor re-stimulation cycles (mean +/- SEM, n=3). ****p < 0.0001 by two-way ANOVA. (**F**) T-cell number was determined prior to each re-stimulation cycle, undertaken as described in C (mean +/- SEM, n = 3). *p < 0.05 by unpaired Student *t*-test.

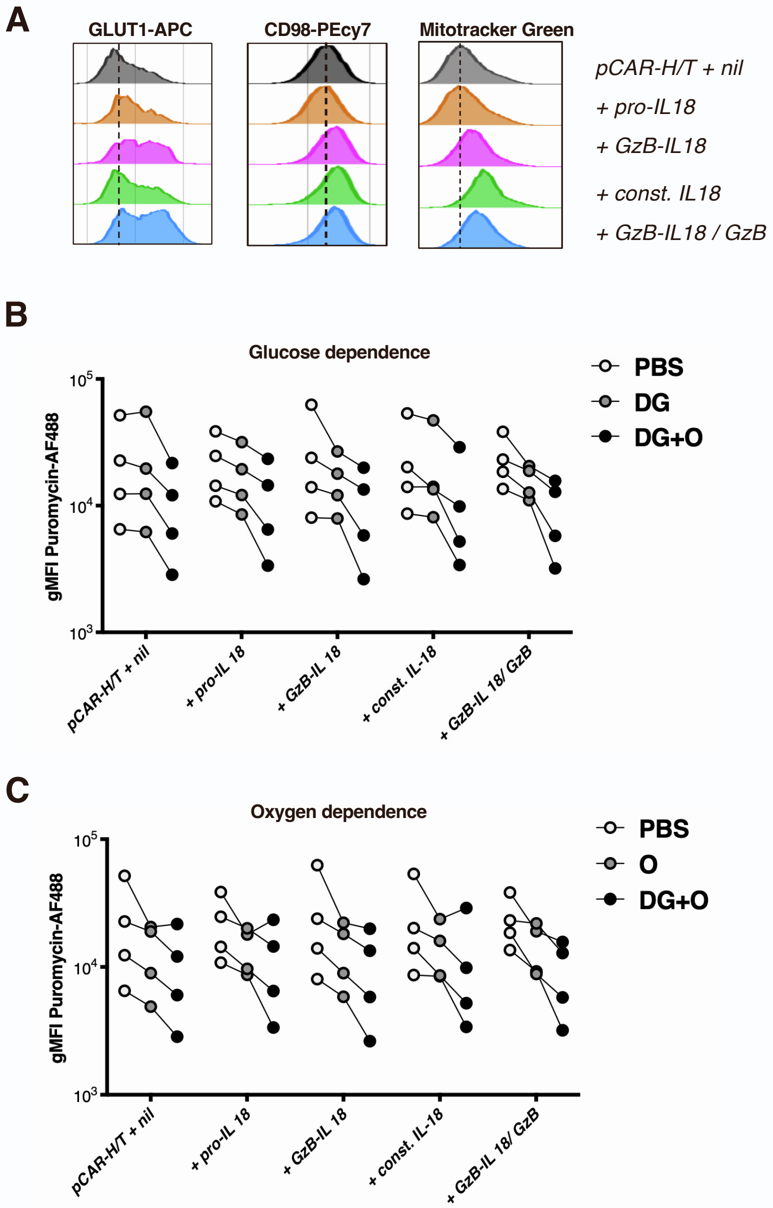

Figure S8. Metabolic analysis of IL18-armored CAR γδ T-cells

(**A**) Representative examples of staining of the indicated markers in *pCAR-H/T* CAR γδ T-cells armored with the specified IL18 variants. Raw data used to calculate glucose dependence (**B**) and oxygen dependence (**C**) using SCENITH are shown. Staining of puromycin in CAR T-cells is shown following incubation with puromycin and either PBS, 2-deoxy-D-glucose (DG), Oligomycin-A (O) or DG + O combined as per SCENITH protocol. (n=4 donors).

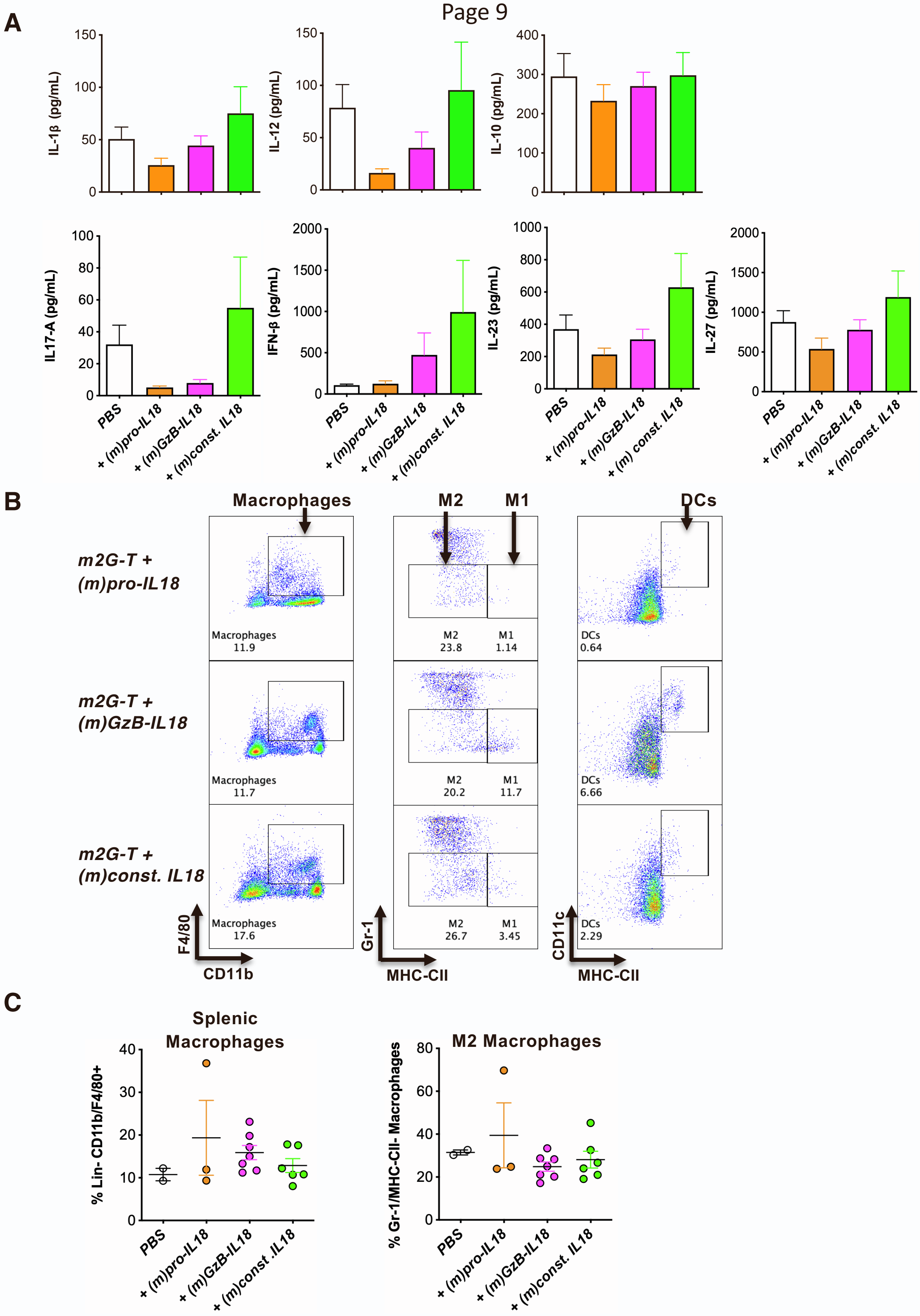

Figure S9. Phenotyping of CAR T-cell treated immunocompetent BALB/c mice
(**A**) Cytokines measured in sera of BALB/c mice engrafted with B7E3 tumors, 24 hours following treatment with *m2G-T* CAR T-cells armored with each murine IL18 variant cycles (mean +/- SEM, n=11-18 mice). All N/S using 2-way ANOVA. (**B**) Gating strategy for phenotyping the spleens of mice described in (A). (**C**) Percentage of macrophages identified as Lin- CD11b+/F4/80+ and M2 macrophages identified as Lin- CD11b+/F4/80+ Gr-1-/MHC-II- cycles (mean +/- SEM, n=2-7 mice). All N/S using one-way ANOVA.
